# CBX1 is involved in hepatocellular carcinoma progression and resistance to sorafenib and lenvatinib via IGF-1R/AKT/SNAIL signaling pathway

**DOI:** 10.1007/s12072-024-10696-0

**Published:** 2024-05-20

**Authors:** Su-Su Zheng, Jing-Fang Wu, Wei-Xun Wu, Jin-Wu Hu, Dai Zhang, Cheng Huang, Bo-Heng Zhang

**Affiliations:** 1 Department of Hepatic Oncology, Xiamen Clinical Research Center for Cancer Therapy, Zhongshan Hospital, Fudan University (Xiamen Branch), Xiamen, 361015 China; 2 Department of Hepatic Oncology, Liver Cancer Institute, Key Laboratory for Carcinogenesis and Cancer Invasion (Ministry of Education), Zhongshan Hospital, Fudan University, 180 Fenglin Road, Shanghai, 200032 China; 3 Department of Liver Surgery, Zhongshan Hospital, Fudan University (Xiamen Branch), Xiamen, 361015 China; 4 Department of Liver Cancer, Shanghai Geriatrics Medical Center, 2560 Chunshen Road, Shanghai, 201104 China; 5 Center for Evidence-Based Medicine, Shanghai Medical School, Fudan University, Shanghai, 200032 China; 6 Department of Liver Surgery, Liver Cancer Institute, Key Laboratory of Carcinogenesis and Cancer Invasion (Ministry of Education), Zhongshan Hospital, Fudan University, Shanghai, 200032 China

**Keywords:** Chromobox Homolog 1, Hepatocellular carcinoma, TKI resistance, AKT signaling, Epithelial−mesenchymal transition

## Abstract

**Background:**

Chromobox Homolog 1 (CBX1) plays a crucial role in the pathogenesis of numerous diseases, including the evolution and advancement of diverse cancers. The role of CBX1 in pan-cancer and its mechanism in hepatocellular carcinoma (HCC), however, remains to be further investigated.

**Methods:**

Bioinformatics approaches were harnessed to scrutinize CBX1’s expression profile, its association with tumor staging, and its potential impact on patient outcomes across various cancers. Single-cell RNA sequencing data facilitated the investigation of CBX1 expression patterns at the individual cell level. The CBX1 expression levels in HCC and adjacent non-tumor tissues were quantified through Real-Time Polymerase Chain Reaction (RT-PCR), Western Blotting (WB), and Immunohistochemical analyses. A tissue microarray was employed to explore the relationship between CBX1 levels, patient prognosis, and clinicopathological characteristics in HCC. Various in vitro assays—including CCK-8, colony formation, Transwell invasion, and scratch tests—were conducted to assess the proliferative and motility properties of HCC cells upon modulation of CBX1 expression. Moreover, the functional impact of CBX1 on HCC was further discerned through xenograft studies in nude mice.

**Results:**

CBX1 was found to be upregulated in most cancer forms, with heightened expression correlating with adverse patient prognoses. Within the context of HCC, elevated levels of CBX1 were consistently indicative of poorer clinical outcomes. Suppression of CBX1 through knockdown methodologies markedly diminished HCC cell proliferation, invasive capabilities, migratory activity, Epithelial−mesenchymal transition (EMT) processes, and resistance to Tyrosine kinase inhibitors (TKIs). Contrastingly, CBX1 augmentation facilitated the opposite effects. Subsequent investigative efforts revealed CBX1 to be a promoter of EMT and a contributor to increased TKI resistance within HCC cells, mediated via the IGF-1R/AKT/SNAIL signaling axis. The oncogenic activities of CBX1 proved to be attenuable either by AKT pathway inhibition or by targeted silencing of IGF-1R.

**Conclusions:**

The broad overexpression of CBX1 in pan-cancer and specifically in HCC positions it as a putative oncogenic entity. It is implicated in forwarding HCC progression and exacerbating TKI resistance through its interaction with the IGF-1R/AKT/SNAIL signaling cascade.

**Supplementary Information:**

The online version contains supplementary material available at 10.1007/s12072-024-10696-0.

## Introduction

Hepatocellular carcinoma (HCC), a common and deadly form of liver cancer, is the second primary cause of cancer-related death globally [1]. Advances in diagnosis and treatment have been made, yet patients’ prognoses remain discouraging, demonstrated by a mere 30% five-year survival rate post-curative surgery [2]. Recurrence and metastasis are major contributors to poor postoperative outcomes, and the complex mechanisms behind these events are not yet fully understood [3]. Unraveling the function of critical molecules in HCC progression could lead to improved therapeutic strategies and enhanced patient survival.

The Chromobox (CBX) family of proteins encompasses eight variants (CBX1-8), each playing a pivotal role in multiple biological endeavors, such as gene regulation and developmental processes [4]. Of particular interest is CBX1, commonly referred to as HP1β, which is integral to embryonic stem cell pluripotency and differentiation [5]. The multifaceted nature of CBX1 is evident in its regulatory functions, which involve subcellular positioning, protein interactions, and chromatin association—all of which vary across developmental stages [6]. Notably, CBX1 is implicated in embryonic murine neurogenesis and brain formation [7]. Its involvement extends to cellular proliferation; for example, it's regulated by miR-205-5p in pituitary tumors and has been identified in HCC to promote cell growth via the Wnt/β-Catenin pathway [8, 9]. Elevated CBX1 expression, associated with poor clinical outcomes, has been documented in cancers like breast and ovarian [10, 11]. Yet, CBX1’s comprehensive role in pan-cancer scenarios and its specific mechanistic contribution to HCC are not fully characterized.

Our multifaceted study, encompassing bioinformatic evaluations, immunohistochemistry, tissue microarray assessments, and both in vitro and in vivo experimental analysis, establishes the pronounced upregulation of CBX1 across majority of cancer types, including HCC. This upregulation correlates with aggressive tumor behaviors, positioning CBX1 as an independent indicator of unfavorable prognosis for HCC patients. Functional studies reveal CBX1 as a facilitator of HCC cell proliferation, invasion, migration, and EMT, as well as an enhancer of resistance to Tyrosine Kinase Inhibitors (TKIs) through the IGF-1R/AKT/SNAIL signaling pathway. Both AKT inhibitors and IGF-1R ablation can effectively mitigate the tumorigenic influences of CBX1.

With this evidence, CBX1 emerges not only as a marker of tumoral virulence but also as a prospective target for therapeutic intervention, cementing its significance in the future of HCC treatment.

## Materials and methods

### Data source

The expression of CBX1 in pan-cancer was obtained from the Gene_DE module of TIMER2 (http://timer.cistrome.org/). The protein expression of CBX1 in selected tumors was obtained from The UALCAN portal (http://ualcan.path.uab.edu/analysis-prot.html). The correlation between CBX1 and pan-cancer tumor staging was analyzed using GEPIA2 (http://gepia2.cancer-pku.cn/#analysis). Gene expression levels were represented as log2 [TPM (Transcripts per million) + 1]. The prognostic effect of CBX1 in pan-cancer was determined using the Survival Analysis module of GEPIA2. Single-cell RNA sequencing (ScRNA-seq) data from GSE112271 were utilized to examine the expression of CBX1 in different cell types [12]. Gene set enrichment analysis (GSEA) was performed to identify potential signaling pathways involving CBX1 from the cancer genome atlas (TCGA) HCC data (https://portal.gdc.cancer.gov/repository), details for performing GSEA was described previously [13].

### Clinical subjects and follow-up

This study included four cohorts of subjects. Cohort 1 consisted of 30 patients who underwent HCC resection. Tumor specimens were collected in October 2022, and both cancerous and adjacent tissues were preserved for RT-PCR analysis. Cohort 2 comprised 12 patients who underwent HCC resection. Specimens were collected in December 2022, and Western blotting was conducted to evaluate CBX1 protein expression in HCC. Cohort 3 consisted of 208 patients who underwent HCC resection. Specimens were collected between January 2012 and December 2012 for tissue microarray construction and immunohistochemical staining. Cohort 4 comprised 106 patients with unresectable HCC treated with sorafenib. Specimens were collected between January 2015 and December 2016. The diagnosis of HCC was based on the American Association for the Study of Liver Disease guidelines. Tumor staging was determined according to the Barcelona clinic liver cancer (BCLC) criteria [14]. This study was approved by the Ethics Committee of Zhongshan Hospital, Fudan University, and informed consent was obtained from all participants. Patient follow-up continued until December 2018. The time to recurrence (TTR) was defined as the interval between treatment and intrahepatic recurrence or extrahepatic metastasis. Overall survival (OS) was defined as the interval between treatment and death from any cause or the last observation date. Progression-free survival (PFS) was defined as survival without relapse or progression.

### Cell cultivation and cell lines

The cell lines L02, MHCC97H, MHCC97L, HCCLM3, Huh7, and HepG2 were acquired from Fudan University’s Liver Cancer Institute located in Shanghai, while the Hep3B cell line was sourced from the Institute of Biochemistry and Cell Biology’s cell bank under the Chinese Academy of Science, Shanghai. Cultivation conditions for these cells consisted of high-glucose DMEM with a 10% FBS and 1% penicillin–streptomycin mix, within a 5% CO2 atmosphere at 37ºC. Cell culture materials were procured from Gibco by Thermo Fisher Scientific in the United States.

### Transfection procedures

Short hairpin RNA sequences targeting CBX1, Snail, and IGF-1R, were engineered into the pLVX-Puro vector from GeneChem, Shanghai. Viral collections were done at 48 h post-transfection and filtered using 0.45-μM syringe filters before infecting target cells with polybrene at 8 μg/ml. To induce CBX1, Snail, and IGF-1R expression, the pLVX plasmid was synthesized by GeneChem. Infected cells were selected with 1 μg/ml puromycin. Retroviruses are generated by packaging 293 T cells. The sequence of pLKO.1-shRNA targeting the CBX1 mRNA was CCGGCCCGACCTCATTGCTGAGTTTCTCGAGAAACTCAGCAATGAGGTCGGGTTTTTG for shCBX1-1, CCGGCCTCCTAAAGTGGAAGGGATTCTCGAGAATCCCTTCCACTTTAGGAGGTTTTTG for shCBX1-2 and CCGGCCCACAGGTTGTCATATCCTTCTCGAGAAGGATATGACAACCTGTGGGTTTTTG for shCBX1-3.

The sequence of pLKO.1-shRNA targeting the SNAIL1 mRNA was CCGGGCAGGACTCTAATCCAGAGTTCTCGAGAACTCTGGATTAGAGTCCTGCTTTTTG for shSNAIL1-1, CCGGCCAATCGGAAGCCTAACTACACTCGAGTGTAGTTAGGCTTCCGATTGGTTTTTG for shSNAIL1-2 and CCGGCCACTCAGATGTCAAGAAGTACTCGAGTACTTCTTGACATCTGAGTGGTTTTTG for shSNAIL1-3.

The sequence of pLKO.1-shRNA targeting the IGF-1R mRNA was CCGGGCGGTGTCCAATAACTACATTCTCGAGAATGTAGTTATTGGACACCGCTTTTTG for shIGF-1R-1, CCGGGCGGTGTCCAATAACTACATTCTCGAGAATGTAGTTATTGGACACCGCTTTTTG for shIGF-1R-2 and CCGGGCTGTACGTCTTCCATAGAAACTCGAGTTTCTATGGAAGACGTACAGCTTTTTG for shIGF-1R-3.

Coding sequence of human CBX1, Snail1 and IGF-1R was cloned into the pCDH-CMV-MCS-EF1-CopGFP-T2A-Puro vector by mutiple cloning sites [15].

### Utilization of antibodies and chemicals

Antibodies used in this study were anti-CBX1, E-Cadherin, N-Cadherin, Vimentin, Snail, pAKT, AKT, Twist, Slug, Zeb1, p65, phospho-p65, ERK, phospho-ERK, JNK, phospho-JNK, SMAD3, phospho-SMAD3, IGF-1R, IGF-1, EGFR, VEGFR1, FGFR1, KIT, PDGFR-α, TGFβ-R1, all sourced from Abcam; and MK-2206 (an AKT inhibitor), SC-79 (an AKT activator) from Selleck.

### Immunohistochemistry and tissue microarray analysis

Tissue microarrays (TMAs) with two HCC cohorts were prepared as previously detailed [16]. Breifly, slides were treated with primary antibodies against human CBX1 and left to incubate at 4℃ throughout the night after undergoing rehydration and antigen retrieval via microwave heating. This was followed by a 30 min incubation with secondary antibodies at a temperature of 37 ℃. The staining process involved the application of 3′3-diaminobenzidine tetrahydrochloride, and was completed with a counterstain using Mayer’s hematoxylin. Two independent pathologists carried out the evaluation of the immunohistochemical staining intensity, and any differences in assessment were reconciled through joint agreement. Antibody dilution was optimized against normal tissue controls for maximal sensitivity and specificity. TMA staining employed the EnVision + system and diaminobenzidine, excluding primary antibodies for negative controls. The immunoreactive score was based on the extent (0–4) and intensity (0–3) of staining, yielding an overall score between 0 and 12, with low (0–6) and high (8–12) categories [17]. Independent pathologists, blinded to patient data, conducted evaluations, resolving any inconsistencies by consensus.

### Quantitative real-time PCR

Total RNA extraction and cDNA synthesis were performed using kits from Qiagen, with gene quantification via FastStart Universal SYBR Green Master on a LightCycler 480 system, both from Roche Diagnostics, following manufacturer guidelines. Gene expression normalization was against GAPDH, employing the ΔCq method with a PCR protocol of an initial 95 °C for 5 min, followed by 40 cycles of 95 °C for 10 s and 60 °C for 60 s. The equation is as follows: 2^−ΔCt^ (ΔCt = Ct[target gene]-Ct[GAPDH]). Primers used in this study were list in supplementary Table 1.

### Protein analysis by western blotting (WB)

Cell lysates were prepared using RIPA buffer with PMSF, then centrifuged, and protein concentrations determined via BCA assay. Proteins were denatured, resolved on SDS-PAGE, and transferred to PVDF membranes. Incubation with primary and secondary antibodies was followed by detection, with GAPDH as the loading control. Densitometry was employed for quantitative analysis using NIH Image J software, with normalization to GAPDH and control conditions.

### Cell proliferation assessment

Cell proliferation was assessed using Cell Counting Kit-8(CCK-8) assays at 24, 48, and 72 h, following the protocol provided by Dojindo. Absorbance at 450 nm indicated cell viability. Colony formation was evaluated over 14 days post-seeding at 1000 cells per well, with 4% paraformaldehyde fixation and Giemsa staining, followed by manual colony counting.

### Invasion assays

Transwell assays with Matrigel coating assessed invasion, seeding 5 × 104 cells in the upper chamber, and using DMEM with 10% FBS as an attractant. After 24 h, migrated cells were fixed, stained, and counted under a microscope. Each experiment was replicated three times, with statistical analysis via the Student’s *t* test.

### Wound healing assay

This assay followed standardized methods, with cells incubated in DMEM plus 1% FBS post-PBS wash. All procedures were replicated thrice.

### Cignal finder RTK reporter array

We utilized the Cignal Finder RTK Reporter Array from Qiagen, Germany, to explore the signaling pathways that CBX1 influences in hepatocellular carcinoma (HCC) cell lines. Cells in a logarithmic phase of growth were seeded at a density of 1 × 10^5 cells per well in 96-well plates and allowed to adhere for 16 h. These cells were then transfected with reporter constructs responsive to various transcription factors indicative of multiple signaling pathways. After transfection, the original medium was replaced with a fresh one containing 0.5% fetal bovine serum (FBS) and 1% antibiotic mixture. To assess the impact of CBX1 on HCC, we carried out a luciferase reporter assay employing the dual-luciferase reporter assay system by Promega, headquartered in Wisconsin, USA, adhering to the manual’s guidelines. This involved adding 100 μL of Dual-Glo Reagent to each well post-incubation to facilitate cell lysis over a 10 min period. Firefly luminescence readings were then acquired with a luminometer. This step was followed by the addition of 100 μL of $Dual-Glo Stop & Glo $Reagent to each well, and after a further 10 min incubation, Renilla luminescence was recorded. The luminescence readings of the experimental group were normalized to those of the control, and the relative response ratios were calculated using these normalized values [18].

### Enzyme-linked immunosorbent assay (ELISA)

The levels of IGF1 present in the supernatant of cultured HCC cells were quantified utilizing an ELISA. We procured the necessary ELISA kits from Abcam, UK, to conduct these measurements. Each assay was performed in triplicate to ensure the reliability of the results.

### Xenograft studies and therapeutic interventions

Immunodeficient BALB/c nude mice from the Chinese academy of medical science were used to establish a xenograft model, as detailed in prior studies [19]. 5 × 10^6^ cancer cells were injected subcutaneously into the ventral area of nude mice, and mice were euthanized 5 weeks post-implantation. The tumor size was measured using calipers. For TKI therapy, mice with established xenografts were divided into treatment groups, and tumor volume was monitored after 5 weeks.

### Statistical analysis

Differences between groups were analyzed using either the Student’s *t* test or Mann–Whitney test, while the Pearson’s correlation test was utilized to examine the association between CBX1 expression and clinicopathological parameters. Survival analysis was performed using the Kaplan–Meier method. Prognostic factors were identified through univariate and multivariate Cox analyses. Statistical significance was set at a *p* value < 0.05 if not specified. All data analyses were conducted using R software (version 4.1.3).

## Results

### Expression of CBX1 across multiple cancer types

**Fig. 1:**
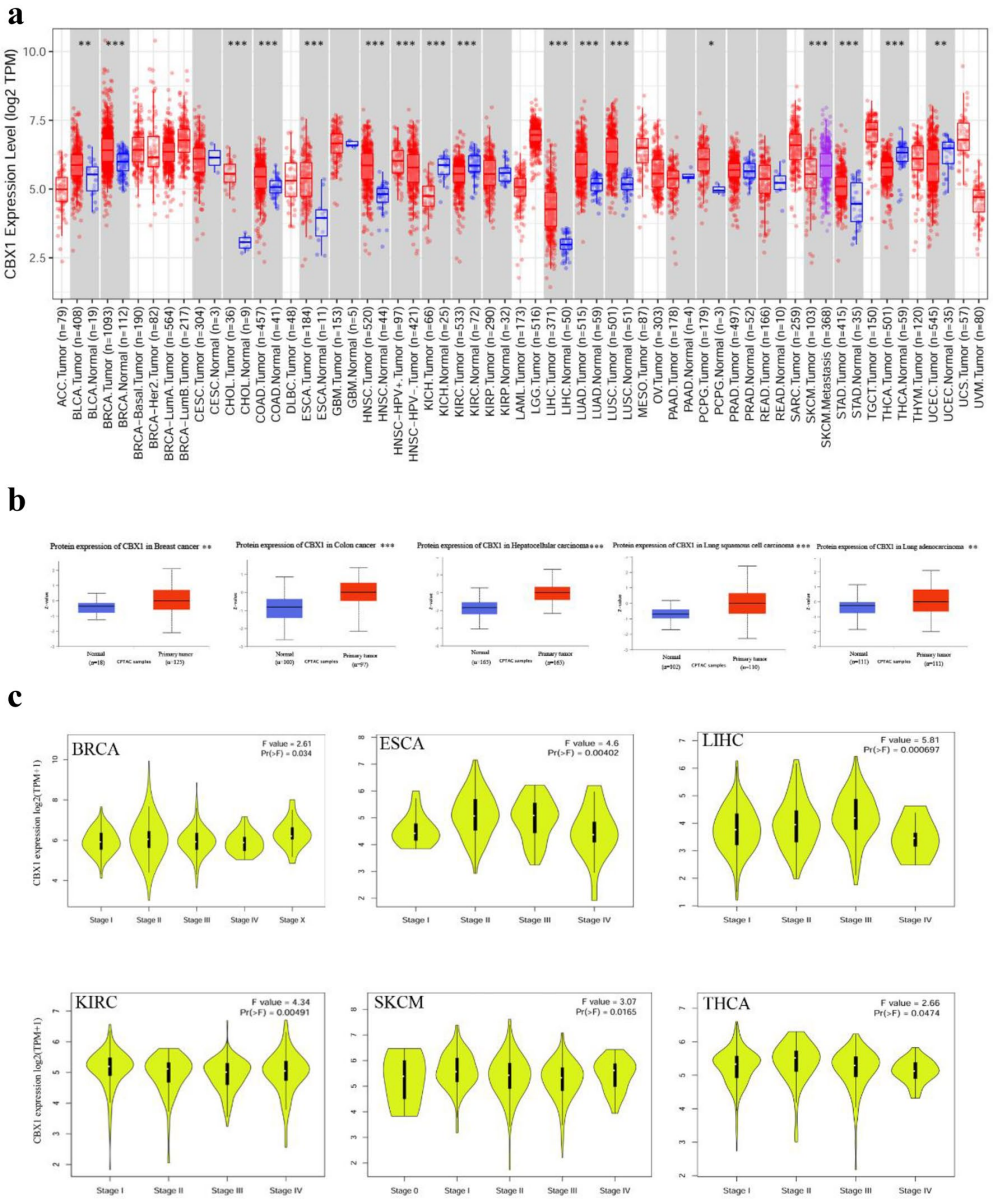
CBX1 Expression Across Tumor Types and Stages. **A** TIMER2 database analysis showing CBX1 gene expression across various tumor types. **B** CPTAC dataset analysis depicting CBX1 protein levels in breast, colon, HCC, lung squamous cell carcinoma and lung adenocarcinoma. **C** TCGA dataset representation of CBX1 expression across different tumor types and stages, with statistical significance markers. For all the figures, *p < 0.05, ** *p* < 0.01, *** p < 0.001

We began by examining CBX1 expression in a range of cancers using the TIMER2.0 web resource, which analyzes gene expression via TCGA data. Figure 1A illustrates that CBX1 is significantly overexpressed in tumor tissues of BLCA (Bladder urothelial carcinoma), BRCA (Breast invasive carcinoma), CHOL (Cholangiocarcinoma), COAD (Colon adenocarcinoma), ESCA (Esophageal carcinoma), HNSC (Head and neck squamous cell carcinoma), LIHC (Liver hepatocellular carcinoma), LUAD (Lung adenocarcinoma), LUSC (Lung squamous cell carcinoma), PCPG (Pheochromocytoma and paraganglioma), and STAD (Stomach adenocarcinoma) compared to adjacent non-tumor tissues (*p* < 0.05 for all). Complementary protein expression analysis from the CPTAC (Clinical proteomic tumor analysis consortium) dataset confirmed heightened CBX1 levels in breast, colon, and liver cancers, as well as lung squamous cell carcinoma and lung adenocarcinoma (*p* < 0.05; Fig. 1B). Utilizing GEPIA2’s Pathological Stage Plot, we observed a correlation between elevated CBX1 expression and advanced clinical stages in BRCA, ESCA, LIHC, KIRC (Kidney renal clear cell carcinoma), SKCM (Skin cutaneous melanoma), and THCA (Thyroid carcinoma) (Fig. 1C). Interestingly, we observed that the correlation between elevated CBX1 expression and advanced clinical stages could not be fully confirmed in ESCA and LIHC, as the expression levels appeared to decrease at stage IV. These phenomena warrant further experimental validation.

### Pan-cancer survival analysis

**Fig. 2:**
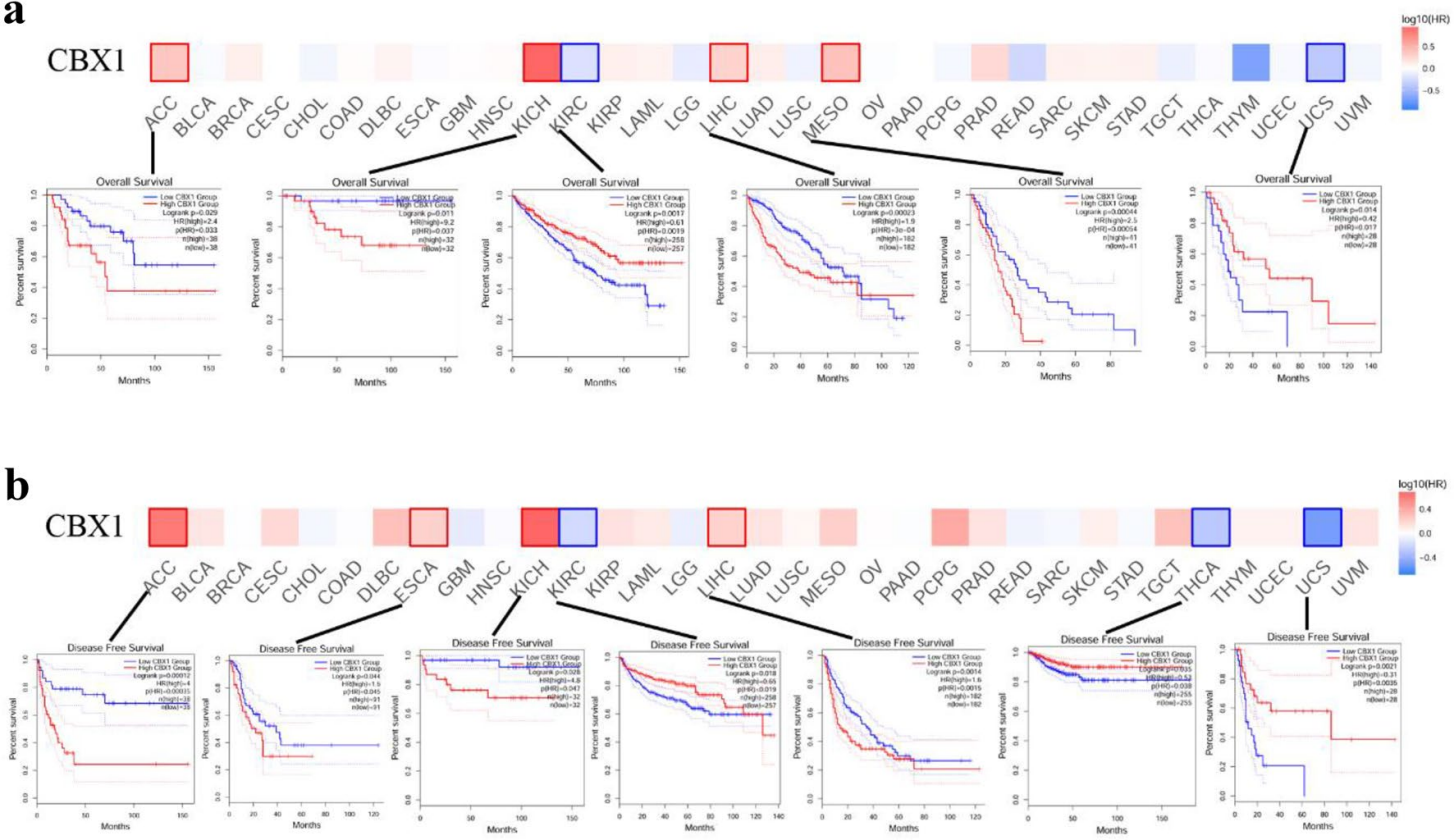
CBX1 Expression’s Relationship with Tumor Prognosis. **A** GEPIA2 tool analysis correlating CBX1 expression with overall survival across different tumors. **B** Graph showing the association between CBX1 expression and disease-free survival in various tumors. Only significant correlations displayed

We assessed the prognostic significance of CBX1 levels using GEPIA2’s Survival Analysis module. High CBX1 expression correlated with reduced overall survival in Adrenocortical carcinoma (ACC), Kidney chromophobe (KICH), KIRC, LIHC, Mesothelioma (MESO), and Uterine carcinosarcoma (UCS). Intriguingly, in KIRC and UCS, elevated CBX1 levels were linked with longer survival. Disease-free survival analysis also showed a negative impact of high CBX1 expression in ACC, ESCA, KICH, and LIHC, whereas a positive trend was noted in KIRC, THCA, and UCS (all *p* < 0.05; Fig. 2A/B). Collectively, these data suggest that CBX1’s aberrant expression across various cancers correlates with patient prognosis.

### CBX1’s role in HCC

**Fig. 3:**
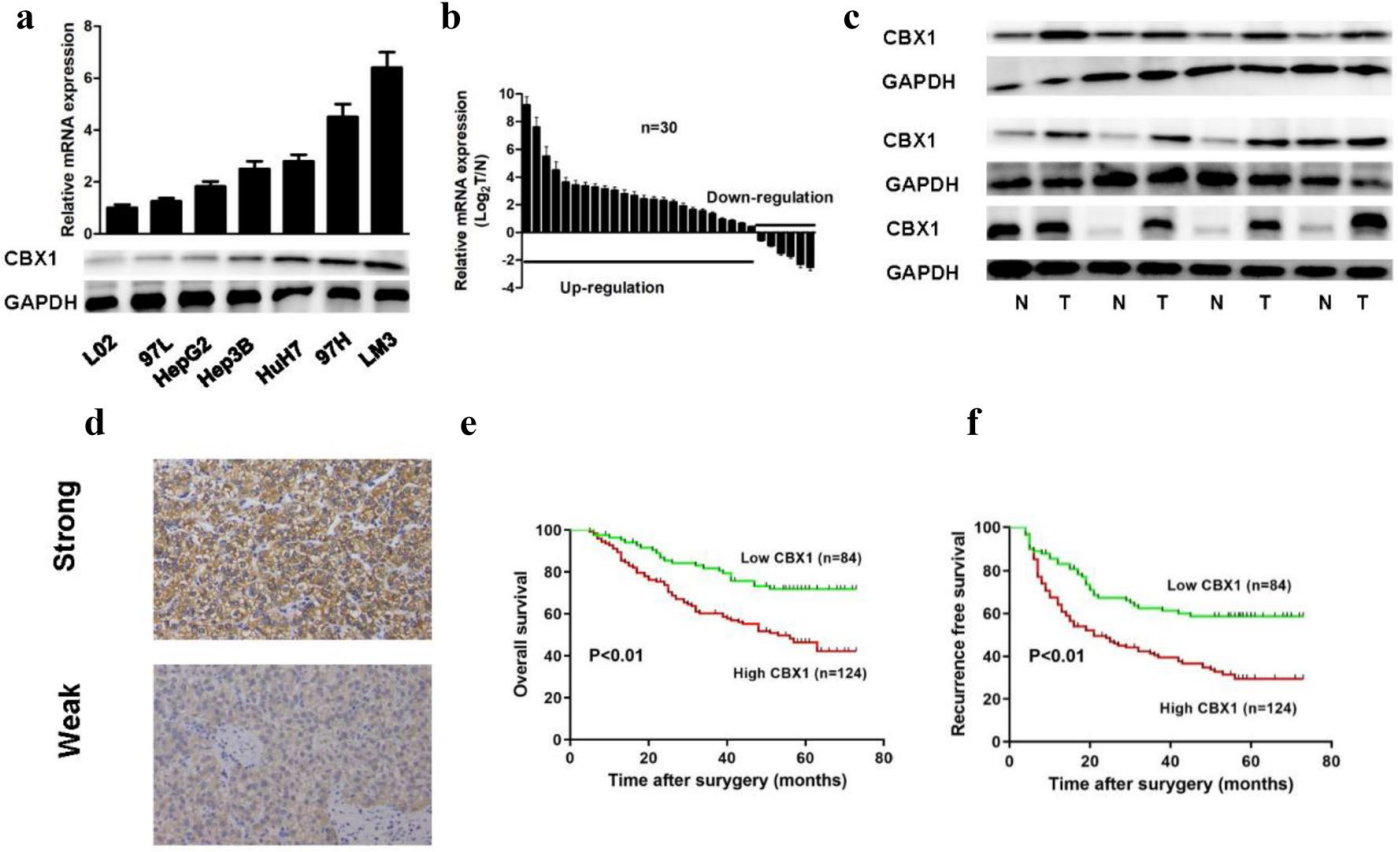
CBX1 in Hepatocellular Carcinoma. **A** CBX1 mRNA and protein levels in HCC cell lines. **B** Comparison of CBX1 mRNA in HCC tumors versus adjacent non-tumor tissue. **C** Protein expression comparison of CBX1 in HCC tumors and adjacent normal tissues. **D** Representative immunohistochemistry staining of HCC tissue microarray showing weak and strong signals. **E** Kaplan–Meier plots for overall survival in HCC patients stratified by CBX1 expression. **F** Disease-free survival Kaplan–Meier plots for HCC patients based on CBX1 levels

Aligning with prior observations, CBX1 is upregulated in HCC (referencing TCGA data) and associates with poor outcomes. Single-cell RNA-seq analysis indicated predominant CBX1 expression in HCC cells and cancer-associated fibroblasts (CAF), the details for conducting single-cell RNA-seq analysis were described in our previous study (Figure S1)[20]. Experimental validation showed CBX1 upregulation in HCC cell lines, especially in those with high metastatic potential like HCCLM3/MHCC97H (Fig. 3A). WB and RT-PCR analyses corroborated these findings in HCC versus adjacent normal tissues (Figs. 3B/C). In the Human Protein Atlas (HPA) database, CBX1 is indicated to have a low expression level in liver tissue (Figure S2). Immunohistochemistry on tissue microarrays further validated these results (Fig. 3D). Based on the immunohistochemistry scores, we classified patients into high and low CBX1 expression groups (124 and 84 cases, respectively), finding that high CBX1 expression was significantly associated with shorter overall and disease-free survival (*p* < 0.01; Figs. 3E/F). In addition, high CBX1 expression closely correlated with elevated AFP levels, larger tumor size, microvascular invasion, and advanced BCLC staging (all *p* < 0.05; Table 1). Multivariate Cox regression identified both CBX1 expression and tumor size as independent risk factors for overall and recurrence-free survival in HCC (all *p* < 0.05; Table 2).

**Table 1:** Correlation analyses between CBX1 and clinicopathological parameters in HCC

Clinical characteristics	CBX1 low (*n* = 84)	CBX1 high (*n* = 124)	*p*
Age, years			
≤ 50	37	56	0.874
> 50	47	68	
Sex			
Female	16	14	0.118
Male	68	110	
HBsAg			
Negative	14	24	0.623
Positive	70	100	
Liver cirrhosis			
No	9	13	0.958
Yes	75	111	
ALT, U/L			
≤ 40	52	60	0.055
> 40	32	64	
AFP, ng/ml			
≤ 400	63	73	**0.016**
> 400	21	51	
Tumor number			
Single	70	105	0.795
Multiple	14	19	
Tumor size, cm			
≤ 5	63	66	**0.001**
> 5	21	58	
Tumor encapsulation			
Complete	42	60	0.819
None	42	64	
Micro vascular invasion			
No	72	80	**0.001**
Yes	12	44	
Edmondson stage			
I-II	65	84	0.130
III-IV	19	40	
BCLC stage			
0 + A	66	81	**0.039**
B + C	18	43	

Bold represent the p-values less than 0.05 to highlight the variables with statistically significant differences

*CBX1* Chromobox 1, *ALT* alanine aminotransferase, *AFP* α-fetoprotein, *BCLC* barcelona clinic liver cancer

**Table 2:** Multivariable regression analysis of independent prognostic factors in HCC

Variables	Recurrence		Overall survival	
	HR (95% CI)	*p*	HR (95% CI)	*p*
Tumor number (multi versus single)	1.48 (0.91–2.41)	0.114	1.28 (0.74–2.20)	0.381
Tumor size, cm (> 5 versus ≤ 5)	1.74 (1.18–2.58)	**0.006**	1.71 (1.10–2.67)	**0.017**
Micro vascular invasion (Yes versus No)	1.27 (0.83–1.96)	0.276	1.55 (0.96–2.51)	0.073
Edmondson stage (III-IV versus I-II)	1.41 (0.95–2.10)	0.092	1.51 (0.96–2.37)	0.074
CBX1 (high versus low)	1.83 (1.21–2.77)	**0.004**	1.79 (1.09–2.94)	**0.021**

Bold represent the p-values less than 0.05 to highlight the variables with statistically significant differences

*CBX1* Chromobox 1, *HR* hazard ratio

### In vitro CBX1-driven HCC progression

**Fig. 4:**
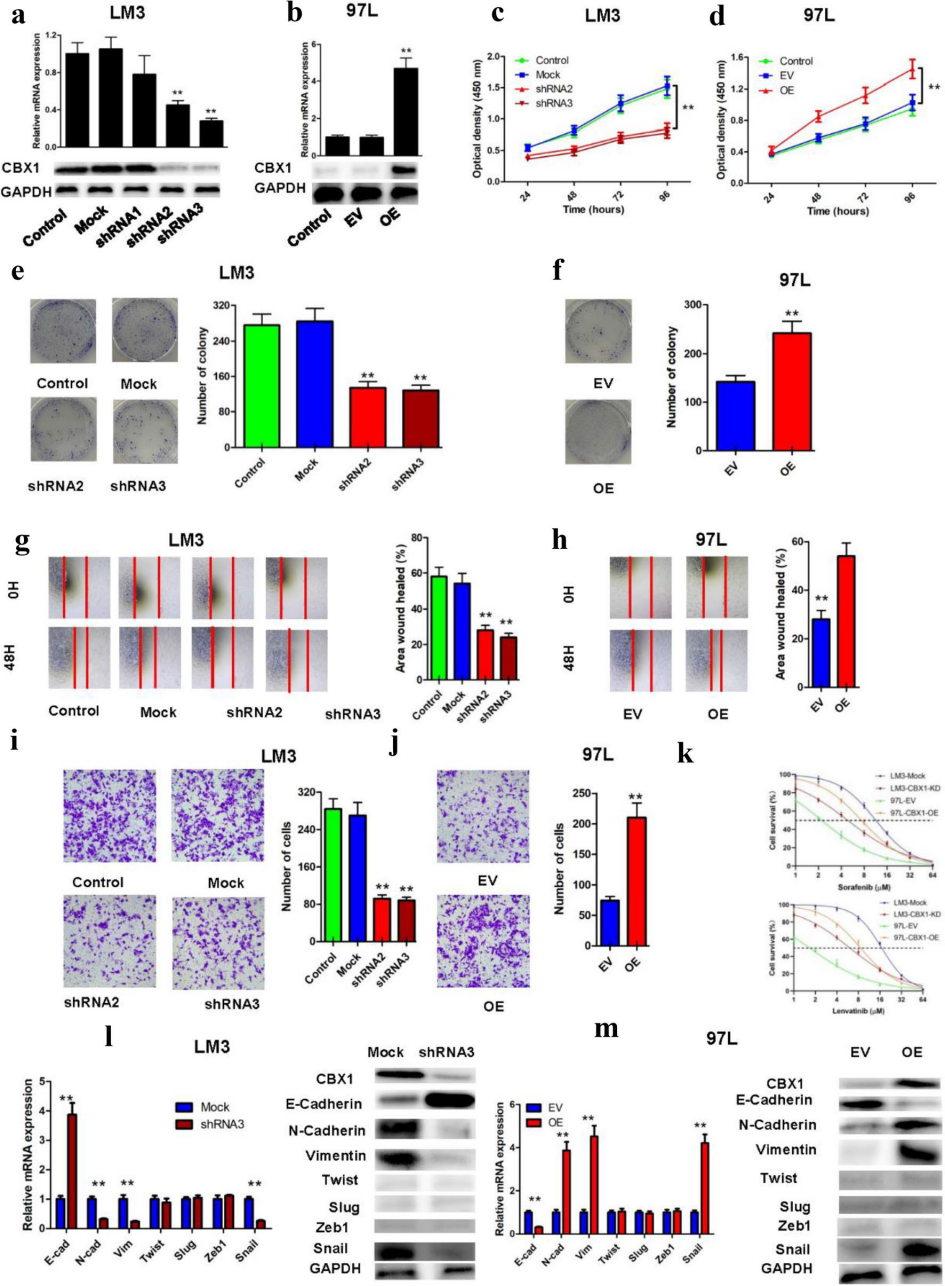
In vitro impact of CBX1 on HCC Progression. **A** CBX1 knockout validation in HCCLM3 cells. **B** Confirmation of CBX1 overexpression in MHCC97L cells. **C** Cell proliferation assay after CBX1 gene knockout in HCCLM3 cells. **D** Cell proliferation assay after CBX1 gene overexpression in MHCC97L cells. **E** Colony-forming assay after CBX1 gene knockout in HCCLM3 cells. **F** Colony-forming assay after CBX1 gene overexpression in MHCC97L cells. **G** Scratch assay after CBX1 gene knockout in HCCLM3 cells. **H** Scratch assay after CBX1 gene overexpression in MHCC97L cells. **I** Transwell invasion assay after CBX1 gene knockout in HCCLM3 cells. **J** Transwell invasion assay after CBX1 gene overexpression in MHCC97L cells. **K** CCK8 assays show TKI sensitivity in CBX1 knockout or overexpression cells. **L** Expression of EMT-related markers in HCCLM3 cells after CBX1 gene knockout. **M** Expression of EMT-related markers in MHCC97L cells after CBX1 gene overexpression. EV, empty vector

Manipulating CBX1 in HCC cell lines through overexpression or shRNA-mediated knockdown yielded conclusive results. In high CBX1-expressing HCCLM3 cells, shRNA2 and shRNA3 effectively suppressed CBX1, as confirmed by RT-PCR and WB (Fig. 4A). Conversely, CBX1 overexpression in MHCC97L cells was also verified (Fig. 4B). Similarly, the expression of CBX1 was knocked down in MHCC97H cells and overexpressed in HepG2 cells, which was verified by RT-PCR and WB (Figure S7A/B). CBX1 knockdown resulted in notably reduced cell proliferation, shown by CCK-8 and colony formation assays (Figs. 4C/E, Figure S7C/E). Conversely, CBX1 overexpression stimulated proliferation (Figs. 4D/F, Figure S7D/F). Scratch assays demonstrated impaired migration in CBX1-knockdown cells and enhanced migration upon CBX1 overexpression (Figs. 4G/H). Transwell invasion assays further supported these findings (Figs. 4I/J, Figure S7G/H). In addition, the cells with reduced CBX1 expression were more responsive to sorafenib or lenvatinib (Fig. 4K, Figure S7I). The relationship between CBX1 and epithelial−mesenchymal transition (EMT) was also investigated, revealing that CBX1 knockdown increased E-cadherin and decreased N-cadherin, Vimentin, and Snail expression, with the reverse results for CBX1-overexpressing cells (Figs. 4L/M, Figure S7J/K). Hence, CBX1 appears to drive HCC cell proliferation, invasion, migration, and EMT.

### In vivo CBX1-driven HCC progression and EMT

**Fig. 5:**
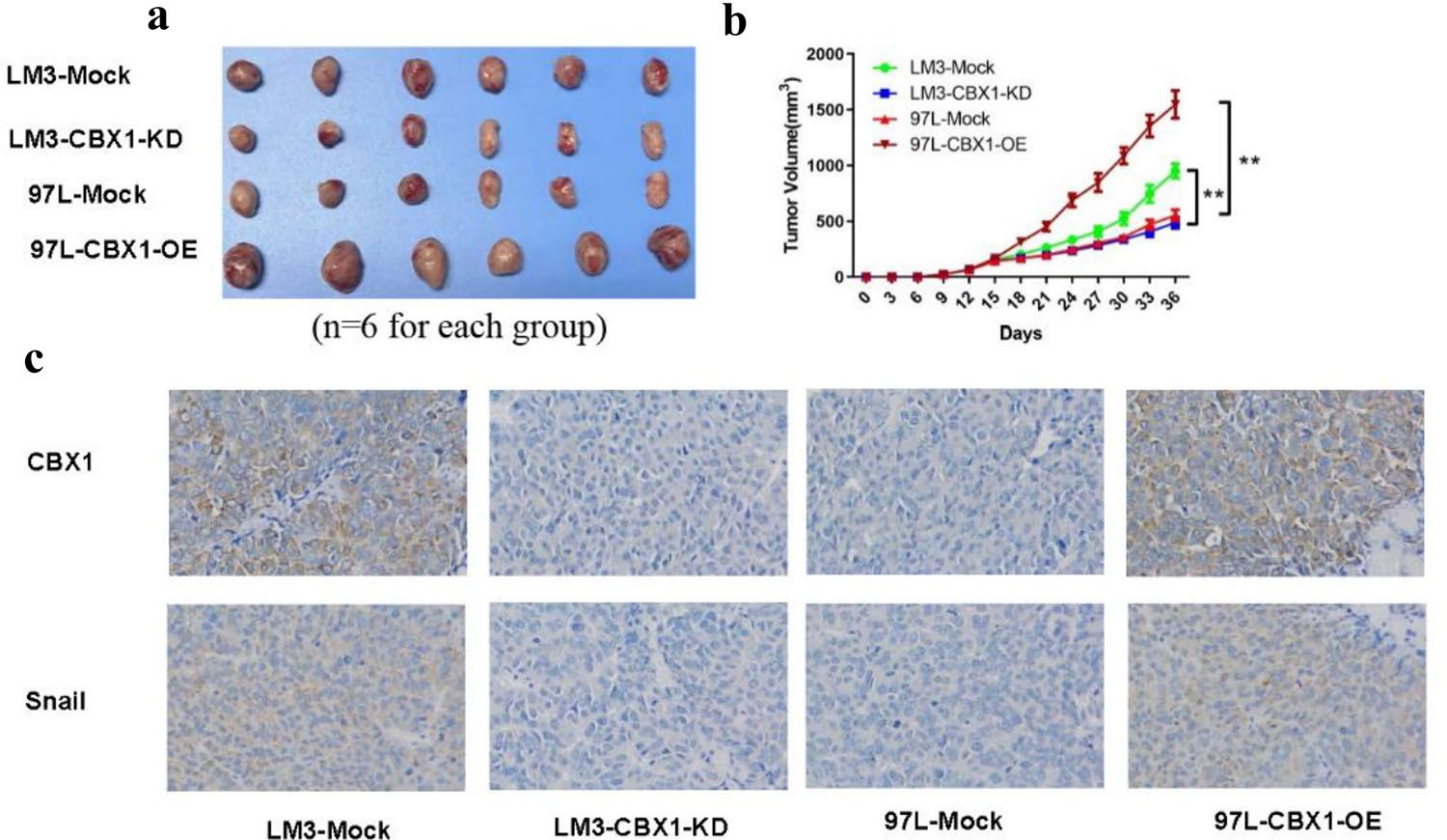
In vivo effects of CBX1 on HCC Tumorigenesis and EMT. **A/B**: Growth of subcutaneous HCC xenografts with altered CBX1 expression after 5 weeks. **C** Snail protein expression by immunohistochemistry in xenografts with different CBX1 levels

CBX1-shRNA3, due to its superior knockdown efficiency, was used for in vivo studies. In a BALB/c mouse xenograft model, CBX1 overexpressing tumors were larger, and immunohistochemical analysis showed increased Snail expression. Conversely, CBX1 knockdown resulted in reduced tumor growth and Snail expression (Figs. 5A/B/C, Figure S7L/M).

### CBX1 regulation of HCC aggressiveness via AKT/SNAIL

**Fig. 6:**
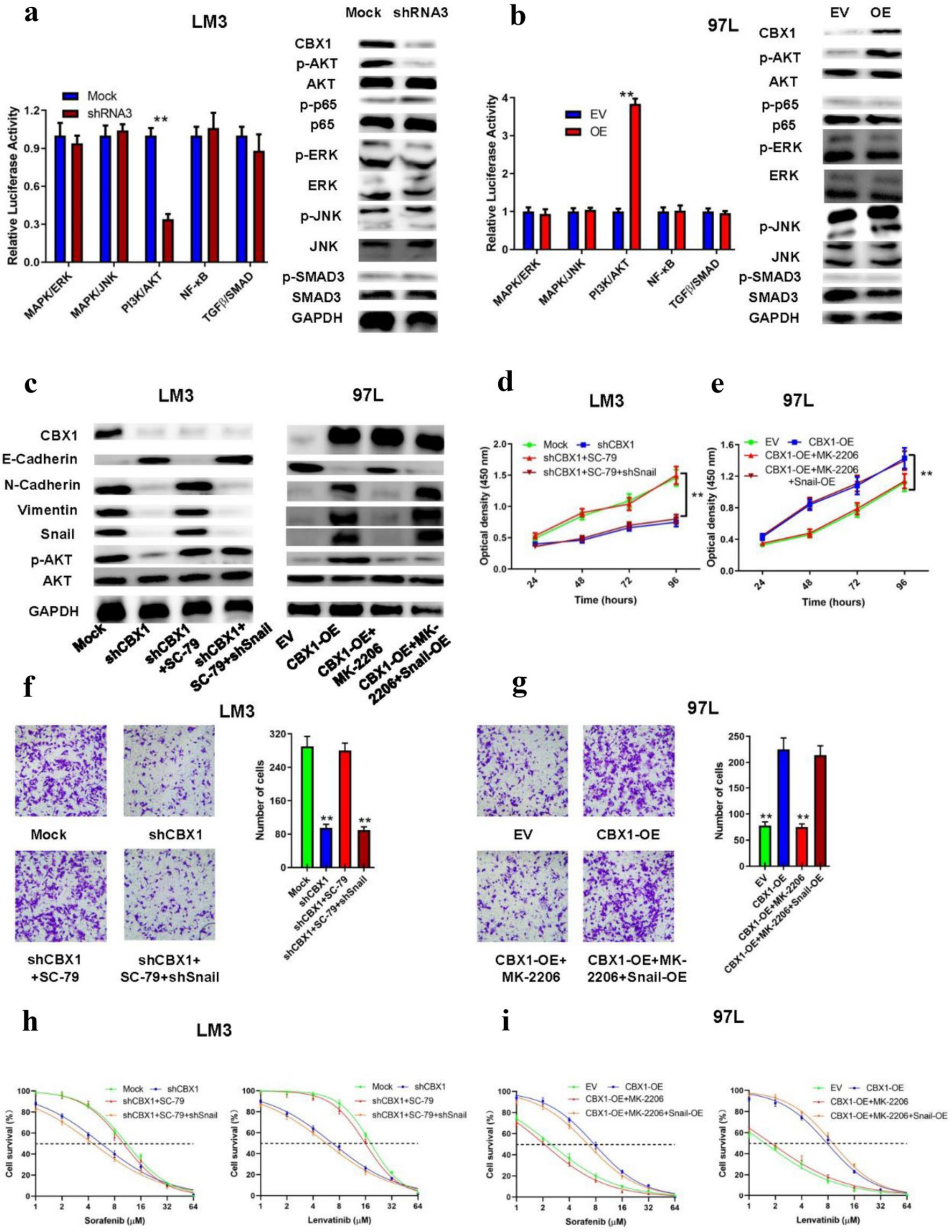
CBX1, EMT, and TKI Resistance via AKT/SNAIL Signaling. **A/B** Reporter assays indicating signaling pathway alterations confirmed by Western blot. **C** EMT marker and AKT protein expression in HCC cells with different CBX1 levels. **D/E** Cell proliferation assay evaluated the proliferation in indicated HCC cells. **F/G** Transwell invasion assay evaluated the invasion ability in indicated HCC cells. **H/I** CCK8 assays showed TKI sensitivity in indicated HCC cells

GSEA of TCGA HCC datasets suggested PI3K/AKT pathway activation in high CBX1 expression groups (Figure S3). Further analysis with the Cignal Finder RTK Reporter Array (QIAGEN, Dusseldorf, Germany) indicated that CBX1 knockdown suppressed, while overexpression activated, the PI3K/AKT pathway, as evidenced by changes in phosphorylated AKT levels (Figs. 6A/B, Figure S8A). WB showed that CBX1 knockdown reduced p-AKT and Snail levels and increased E-cadherin expression, with reversed trends upon AKT activation with SC-79. In contrast, CBX1 overexpression led to increased p-AKT and Snail levels and decreased E-cadherin expression, counteracted by the AKT antagonist MK-2206 (Fig. 6C, Figure S8B). SNAIL overexpression or shRNA-mediated knockdown was confirmed by RT-PCR and WB (Figure S4 A/B). Functional assays confirmed that CBX1 knockdown, AKT inhibition, or SNAIL knockout suppressed HCC cell proliferation and invasion, while their overexpression or activation had the opposite effects (Figs. 6D-G, Figure S8C-F). In addition, the CBX1/AKT/SNAIL axis influenced HCC cell sensitivity to TKIs, with CBX1 knockdown increasing sorafenib and lenvatinib sensitivity, reversible by AKT activation or Snail knockout (Fig. 6H/I, Figure S8G/H).

### Linking CBX1 with HCC TKI Resistance and EMT via IGF-1R/AKT/SNAIL

**Fig. 7:**
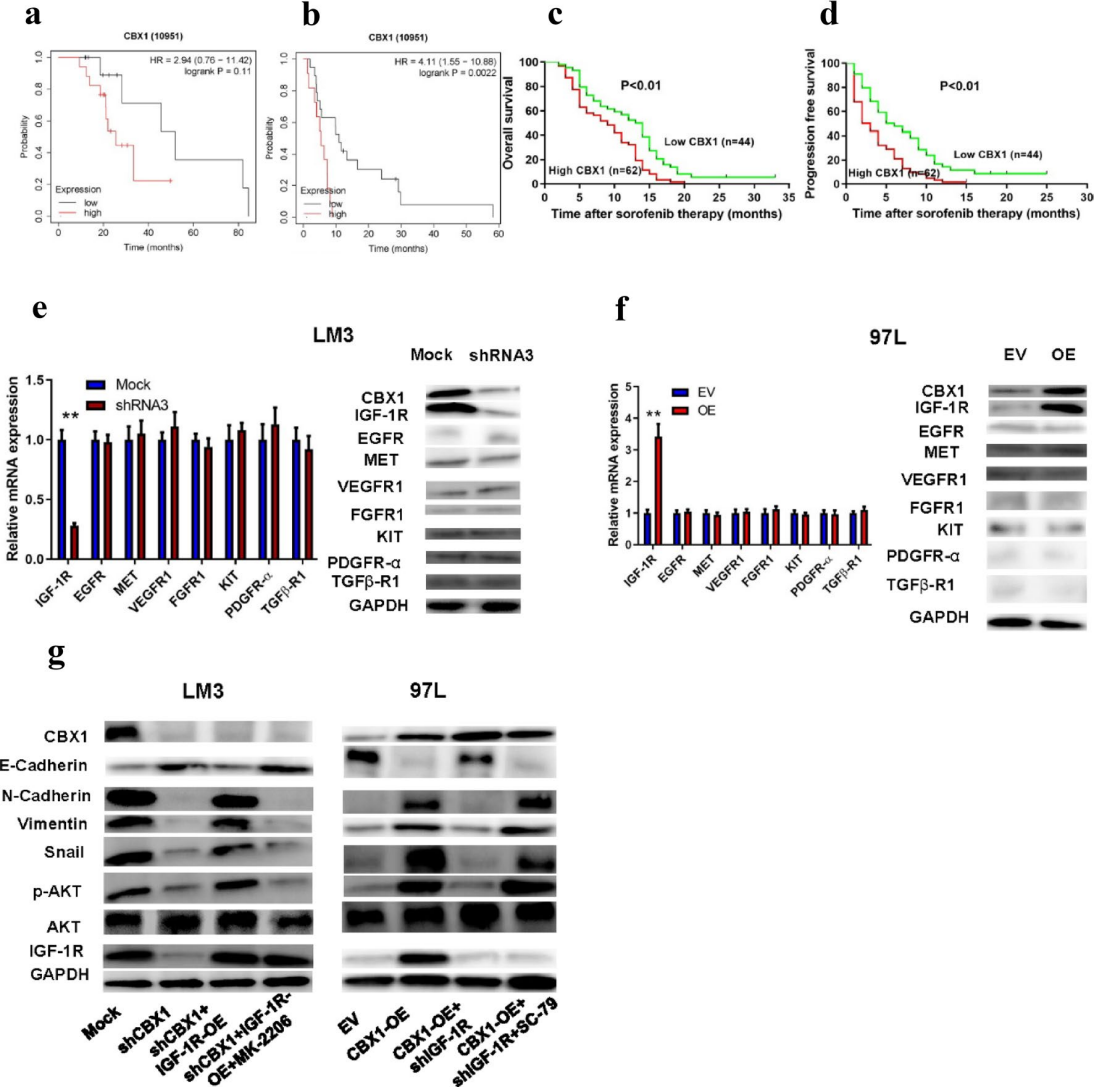
CBX1’s Impact on HCC Response to TKI Therapy. **A** Overall survival of HCC patients in the public database sorafenib treated cohort based on the CBX1 expression. **B** Progression-free survival of HCC patients in the public database sorafenib treated cohort based on CBX1 expression. **C** The overall survival of HCC patients in our sorafenib treated cohort based on CBX1 expression. **D** Progression-free survival of HCC patients in our sorafenib treated cohort based on the CBX1 expression. **E/F** Expression of TKI targeted markers in indicated HCC cells. **G** Expression of CBX1/ IGF-1R/AKT/SNAIL axis related proteins in indicated HCC cells

**Fig. 8:**
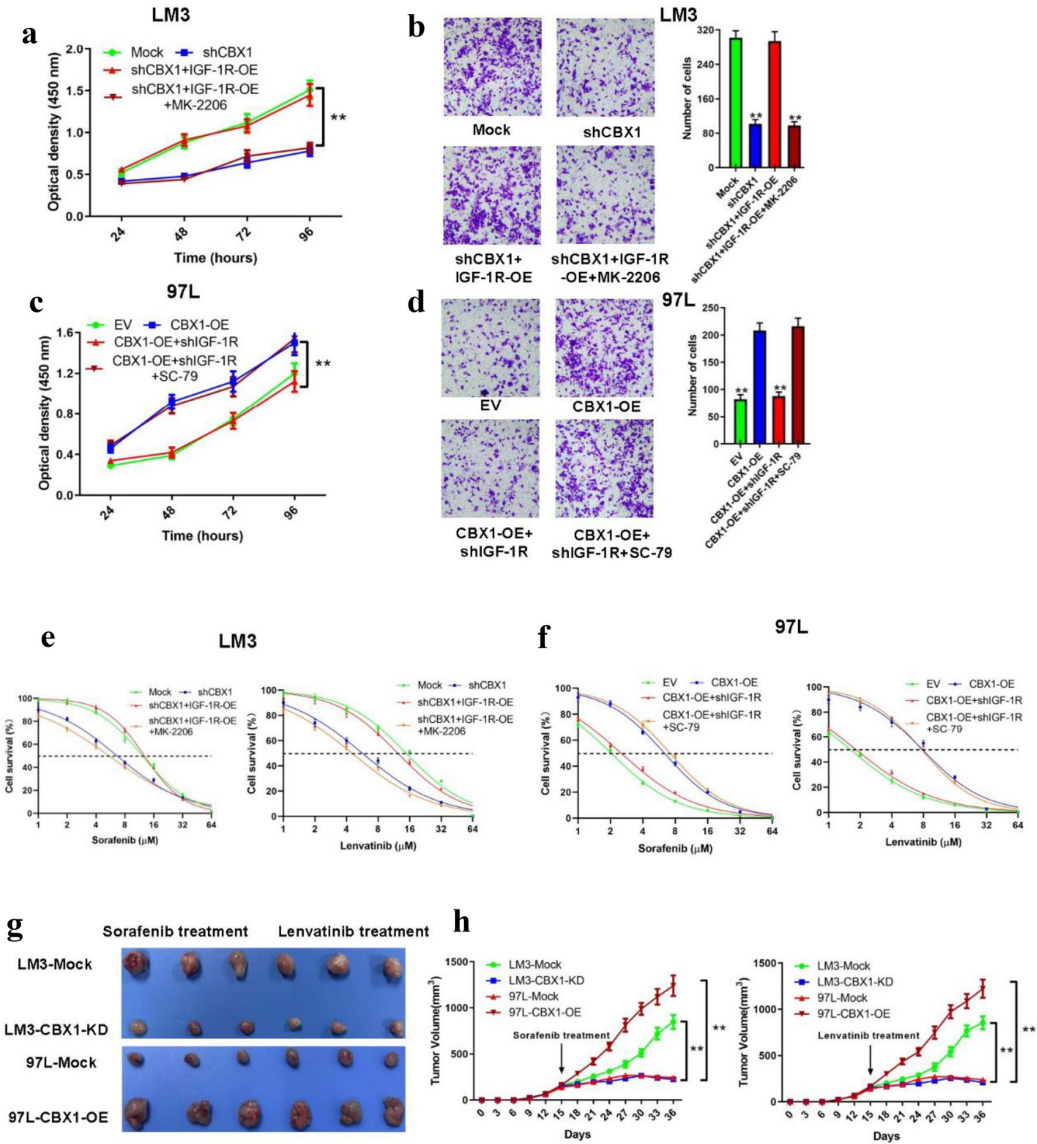
Functional Experiments Demonstrating CBX1/IGF-1R/AKT/SNAIL Axis Promoting HCC Proliferation, Invasion, and TKI Resistance. **A/C** Cell proliferation assays in HCC cells with axis modulation. **B/D** Invasion capability assessment of HCC cells with axis changes. **E/F** TKI sensitivity assays in HCC cells influenced by the axis. **G/H** Xenograft growth and response to sorafenib or lenvatinib in HCC cells with axis alterations, measured over 5 weeks

Exploring CBX1’s impact on sorafenib treatment outcomes, we found high CBX1 expression correlated with shorter progression-free survival in a HCC cohort from The Kaplan–Meier Plotter database (*p* = 0.0022) and suggested a trend towards shorter OS (*p*= 0.11; Figs. 7A/B). A survival study of 106 sorafenib-treated HCC patients from our institution confirmed that high CBX1 expression predicted shorter OS and DFS (*p* < 0.01; Figs. 7C/D). Additionally, high CBX1 expression closely correlated with increased ALT levels, elevated AFP levels, and an increase in the number of tumors (all *p* < 0.05; Table 3). Multivariate analysis identified CBX1, AFP, and microvascular invasion as independent risk factors for recurrence-free survival, with CBX1 and AFP as independent prognostic factor for OS (HR = 1.75, *p* = 0.008; Table 4). Investigating TKI targets (such as IGF-1R, EGFR, MET, VEGFR1, FGFR1, KIT, PDGFR-α, and TGFβ-R1) in CBX1-modified HCCLM3 cells revealed significant IGF-1R downregulation upon CBX1 knockdown (Fig. 7E), with opposite trends in CBX1-overexpressing MHCC97L cells (Fig. 7F). IGF-1R overexpression or shRNA-mediated knockdown was confirmed by RT-PCR and WB (Figure S4 C/D). IGF-1R modulation affected p-AKT, Snail, N-cadherin, and Vimentin expression, which was reversible by MK-2206 or SC-79, suggesting a CBX1/IGF-1R/AKT/SNAIL axis (Fig. 7G). Functional assays indicated that CBX1 and IGF-1R interplay modulated cell proliferation and invasion, and influenced responses to sorafenib and lenvatinib (Figs. 8A-F). In vivo, CBX1 modulation altered tumor response to TKIs, with knockdown enhancing, and overexpression reducing, drug sensitivity (Figs. 8G-H). Additionally, we assessed the expression of IGF1, a common ligand for IGF1R, in HCC cells with either overexpressed or knocked-out CBX1. Results from RT-PCR and ELISA indicated that the alterations in IGF1 levels were not significant in either the overexpression or knockout groups (Figure S5). These data imply that CBX1 fosters HCC TKI resistance, EMT, and progression via the IGF-1R/AKT/SNAIL pathway (Figure S6).

**Table 3:** Correlation analyses between CBX1 and clinicopathological parameters in sorafenib-treated HCC

Clinical characteristics	CBX1 low (*n* = 44)	CBX1 high (*n* = 62)	*p*
Age, years			
≤ 60	21	26	0.554
> 60	23	36	
Sex			
Female	7	12	0.649
Male	37	50	
HBsAg			
Negative	7	9	0.844
Positive	37	53	
Liver cirrhosis			
No	4	8	0.542
Yes	40	54	
ALT, U/L			
≤ 40	26	24	**0.038**
> 40	18	38	
AFP, ng/ml			
≤ 400	25	23	**0.044**
> 400	19	39	
Tumor number			
Single	20	14	**0.013**
Multiple	24	48	
Tumor size, cm			
≤ 5	14	18	0.758
> 5	30	40	
Micro vascular invasion			
No	33	40	0.251
Yes	11	22	

Bold represent the p-values less than 0.05 to highlight the variables with statistically significant differences

*CBX1* Chromobox 1, *ALT* alanine aminotransferase, *AFP* α-fetoprotein

**Table 4:** Multivariable regression analysis of independent prognostic factors in sorafenib-treated HCC

Variables	Disease progress		Overall survival	
	HR (95% CI)	*p*	HR (95% CI)	*p*
AFP, ng/ml (> 400 versus ≤ 400)	1.78 (1.18–2.69)	**0.006**	1.66 (1.11–2.48)	**0.014**
Micro vascular invasion (Yes versus No)	1.66 (1.08–2.57)	**0.022**	1.38 (0.90–2.12)	0.135
CBX1 (high versus low)	1.83 (1.20–2.79)	**0.005**	1.75 (1.15–2.65)	**0.008**

Bold represent the p-values less than 0.05 to highlight the variables with statistically significant differences

*CBX1* Chromobox 1, *HR* hazard ratio

## Discussion

Despite recent advances in the diagnosis and treatment of HCC, the prognosis for patients remains dire, underscoring the urgency to identify and target key molecules involved in HCC proliferation, invasion, and metastasis. Our study contributes to this imperative by establishing CBX1 as a molecule of interest due to its elevated expression in various tumors and its significant association with tumor staging and prognosis, particularly in HCC.

Previous studies have highlighted the important role of CBX1 in maintaining chromosomal stability and gene silencing. Recent research has shown that CBX1 plays a critical role in tumor progression. In nasopharyngeal carcinoma (NPC), CBX1 promotes cell proliferation, invasion, and migration while inhibiting the anti-tumor efficacy of anti-PD-1 drugs [21]. In prostate cancer, CBX1 enhances tumor cell proliferation through the upregulation of androgen receptor [22]. In HCC, one previous study indicated that CBX1 promotes HCC cell proliferation through the Wnt/β-Catenin pathway and is associated with poor prognosis [9]. Our study further expands on these findings by demonstrating that CBX1 is aberrantly expressed in various tumors and is closely associated with aggressive biological behavior and metastasis in HCC.

TKI drugs such as sorafenib and lenvatinib are recommended as first-line treatments for HCC both domestically and internationally [23, 24]. Although immune combination therapy with anti-angiogenic agents is increasingly favored as a preferred treatment option for unresectable HCC, TKIs remain the first choice for patients who cannot receive immunotherapy [25, 26]. However, due to intrinsic or acquired resistance, only approximately 10–30% of patients show response to TKI treatment [27, 28]. Overcoming TKI resistance is therefore of significant clinical importance. EMT plays a vital role in tumor invasion and metastasis [29]. Previous studies have suggested that EMT is one of the important factors leading to TKI resistance. For instance, Mir et al. found that combined inhibition of EMT with sorafenib enhances sensitivity to sorafenib in HCC [30]. Our study demonstrates that CBX1 regulates EMT-related genes and that its knockdown suppresses EMT and TKI resistance in HCC. The AKT signaling pathway is commonly activated in tumor development. The mechanisms of action of TKIs primarily involve the inhibition of RAF/ERK/STAT3 and PI3K/AKT/mTOR signaling pathways [31, 32]. Tan et al. reported that overexpression of TRIM37 promotes AKT activation and confers sorafenib resistance in HCC [33]. Cai et al. revealed that IGF-1R promotes HCC proliferation and anti-apoptosis through the PI3K/AKT signaling pathway [34]. Our findings suggest that high levels of CBX1 can promote the expression of IGF-1R, potentially leading to excessive activation of the IGF-1R/AKT signaling pathway. However, TKI drugs (such as sorafenib) are unable to completely suppress this activation, ultimately resulting in the occurrence of TKI resistance. Thus, CBX1 knockdown can simultaneously suppress EMT and AKT activation, thereby reducing TKI resistance. Moreover, there is currently no consensus on predictive biomarkers for TKI resistance. Our study found that high CBX1 expression in immunohistochemistry staining was associated with worse prognosis in TKI-treated patients, suggesting that routine assessment of CBX1 expression before treatment might help predict TKI efficacy, but further validation is required. While we have identified a correlation between CBX1 and certain downstream effectors, including AKT, we acknowledge the myriad of interactions and feedback mechanisms that also contribute to HCC progression. The above conclusion should still be interpreted with caution.

Our study, however, is not without limitations. The use of nude mice, which lack a complete human tumor microenvironment, may not fully capture the complex interactions in human HCC. Moreover, given the multifaceted nature of TKI resistance and the involvement of the immune system, additional verification in immune-competent animal models is warranted.

## Conclusions

In summary, our study confirms the oncogenic role of CBX1 in HCC. Furthermore, we highlight that high CBX1 expression is an independent prognostic risk factor in HCC and can serve as a prognostic marker. Additionally, we reveal a potential target for TKI resistance and confirm the CBX1/IGF-1R/AKT/SNAIL axis as a potential therapeutic target in HCC.

## Supplementary Information

Below is the link to the electronic supplementary material.

Supplementary file1 (DOCX 5283 KB)

## Data Availability

The data utilized in this research, which have been previously published, can be accessed through the TCGA database (https://portal.gdc.cancer.gov/), TIMER2 (http://timer.cistrome.org/),UALCAN portal (http://ualcan.path.uab.edu/analysis-prot.html), and GEPIA2 (http://gepia2.cancer-pku.cn/#analysis). Any data produced and employed in the course of this study will be made available to interested parties upon request.

## Abbreviations

CBX1: Chromobox Homolog 1
HCC: Hepatocellular carcinoma
RT-PCR: Real-time polymerase chain reaction
WB: Western blotting
CCK-8: Cell counting kit-8
EMT: Epithelial−mesenchymal transition
ELISA: Enzyme-linked immunosorbent Assay
TKIs: Tyrosine kinase inhibitors
ScRNA-seq: Single-cell RNA sequencing
TCGA: The cancer genome atlas
GSEA: Gene set enrichment analysis
BCLC: Barcelona clinic liver cancer
TTR: Time to recurrence
OS: Overall survival
PFS: Progression-free survival
TMAs: Tissue microarrays
BLCA: Bladder urothelial carcinoma
BRCA: Breast invasive carcinoma
CHOL: Cholangiocarcinoma
COAD: Colon adenocarcinoma
ESCA: Esophageal carcinoma
ELISA: Enzyme-linked immunosorbent assay
HNSC: Head and neck squamous cell carcinoma
LIHC: Liver hepatocellular carcinoma
LUAD: Lung adenocarcinoma
LUSC: Lung squamous cell carcinoma
PCPG: Pheochromocytoma and paraganglioma
STAD: Stomach adenocarcinoma
CPTAC: Clinical proteomic tumor analysis consortium
KIRC: Kidney renal clear cell carcinoma
SKCM: Skin cutaneous melanoma
THCA: Thyroid carcinoma
ACC: Adrenocortical carcinoma
KICH: Kidney chromophobe
MESO: Mesothelioma
UCS: Uterine carcinosarcoma
CAF: Cancer-associated fibroblasts
NPC: Nasopharyngeal carcinoma

## References

[1] Sung H, Ferlay J, Siegel RL, Laversanne M, Soerjomataram I, Jemal A, et al. Global cancer statistics 2020: GLOBOCAN estimates of incidence and mortality worldwide for 36 cancers in 185 countries. CA Cancer J Clin. 2021;71:209–249 33538338 10.3322/caac.21660

[2] Dutta R, Mahato RI. Recent advances in hepatocellular carcinoma therapy. Pharmacol Ther. 2017;173:106–117 28174094 10.1016/j.pharmthera.2017.02.010 PMC5777523

[3] Singal AG, Kanwal F, Llovet JM. Global trends in hepatocellular carcinoma epidemiology: implications for screening, prevention and therapy. Nat Rev Clin Oncol. 2023;20:864–884 37884736 10.1038/s41571-023-00825-3

[4] Di Croce L, Helin K. Transcriptional regulation by Polycomb group proteins. Nat Struct Mol Biol. 2013;20:1147–1155 24096405 10.1038/nsmb.2669

[5] Kaufman PD. New partners for HP1 in transcriptional gene silencing. Mol Cell. 2011;41:1–2 21211715 10.1016/j.molcel.2010.12.021 PMC3038578

[6] Nielsen PR, Nietlispach D, Mott HR, Callaghan J, Bannister A, Kouzarides T, et al. Structure of the HP1 chromodomain bound to histone H3 methylated at lysine 9. Nature. 2002;416:103–107 11882902 10.1038/nature722

[7] Mattout A, Aaronson Y, Sailaja BS, Raghu RE, Harikumar A, Mallm JP, et al. Heterochromatin Protein 1beta (HP1beta) has distinct functions and distinct nuclear distribution in pluripotent versus differentiated cells. Genome Biol. 2015;16:213 26415775 10.1186/s13059-015-0760-8 PMC4587738

[8] Hu A, Zhang Y, Zhao X, Li J, Ying Y. CBX1 is a direct target of miR-205-5p and contributes to the progression of pituitary tumor. Pharmazie. 2019;74:154–156 30961681 10.1691/ph.2019.8908

[9] Yang YF, Pan YH, Tian QH, Wu DC, Su SG. CBX1 indicates poor outcomes and exerts oncogenic activity in hepatocellular carcinoma. Transl Oncol. 2018;11:1110–1118 30031230 10.1016/j.tranon.2018.07.002 PMC6074001

[10] Lee YH, Liu X, Qiu F, O’Connor TR, Yen Y, Ann DK. HP1beta is a biomarker for breast cancer prognosis and PARP inhibitor therapy. PLoS ONE. 2015;10: e121207 10.1371/journal.pone.0121207 PMC4358987 25769025

[11] Hu K, Yao L, Xu Z, Yan Y, Li J. Prognostic value and therapeutic potential of cbx family members in ovarian cancer. Front Cell Dev Biol. 2022;10: 832354 35155439 10.3389/fcell.2022.832354 PMC8829121

[12] Losic B, Craig AJ, Villacorta-Martin C, Martins-Filho SN, Akers N, Chen X, et al. Intratumoral heterogeneity and clonal evolution in liver cancer. Nat Commun. 2020;11:291 31941899 10.1038/s41467-019-14050-z PMC6962317

[13] Zheng S, Xie X, Guo X, Wu Y, Chen G, Chen X, et al. Identification of a pyroptosis-related gene signature for predicting overall survival and response to immunotherapy in hepatocellular carcinoma. Front Genet. 2021;12: 789296 34925465 10.3389/fgene.2021.789296 PMC8678488

[14] Forner A, Reig ME, de Lope CR, Bruix J. Current strategy for staging and treatment: the BCLC update and future prospects. Semin Liver Dis. 2010;30:61–74 20175034 10.1055/s-0030-1247133

[15] Hu JW, Yang ZF, Li J, Hu B, Luo CB, Zhu K, et al. TGM3 promotes epithelial−mesenchymal transition and hepatocellular carcinogenesis and predicts poor prognosis for patients after curative resection. Dig Liver Dis. 2020;52:668–676 31822388 10.1016/j.dld.2019.10.010

[16] Jing CY, Fu YP, Yi Y, Zhang MX, Zheng SS, Huang JL, et al. HHLA2 in intrahepatic cholangiocarcinoma: an immune checkpoint with prognostic significance and wider expression compared with PD-L1. J Immunother Cancer. 2019;7:77 30885276 10.1186/s40425-019-0554-8 PMC6421676

[17] Lee JH, Lee JH, Ahn BK, Paik SS, Kim H, Lee KH. Loss of ASXL1 expression is associated with lymph node metastasis in colorectal cancer. Indian J Pathol Microbiol. 2020;63:221–225 32317519 10.4103/IJPM.IJPM_822_19

[18] Hu JW, Yin Y, Gao Y, Nie YY, Fu PY, Cai JB, et al. TM2D1 contributes the epithelial−mesenchymal transition of hepatocellular carcinoma via modulating AKT/beta-catenin axis. Am J Cancer Res. 2021;11:1557–1571 33948373 PMC8085884

[19] Liu WF, Zhang QW, Quan B, Zhang F, Li M, Lu SX, et al. Gas7 attenuates hepatocellular carcinoma progression and chemoresistance through the PI3K/Akt signaling pathway. Cell Signal. 2023;112: 110908 37769891 10.1016/j.cellsig.2023.110908

[20] Zheng SS, Wu YF, Zhang BH, Huang C, Xue TC. A novel myeloid cell marker genes related signature can indicate immune infiltration and predict prognosis of hepatocellular carcinoma: integrated analysis of bulk and single-cell RNA sequencing. Front Mol Biosci. 2023;10:1118377 36959981 10.3389/fmolb.2023.1118377 PMC10027926

[21] Zhao Y, Huang S, Tan X, Long L, He Q, Liang X, et al. N(6) -methyladenosine-modified CBX1 regulates nasopharyngeal carcinoma progression through heterochromatin formation and STAT1 activation. Adv Sci (Weinh). 2022;9: e2205091 36310139 10.1002/advs.202205091 PMC9798977

[22] Itsumi M, Shiota M, Yokomizo A, Kashiwagi E, Takeuchi A, Tatsugami K, et al. Human heterochromatin protein 1 isoforms regulate androgen receptor signaling in prostate cancer. J Mol Endocrinol. 2013;50:401–409 23536649 10.1530/JME-13-0024

[23] Bruix J, Raoul JL, Sherman M, Mazzaferro V, Bolondi L, Craxi A, et al. Efficacy and safety of sorafenib in patients with advanced hepatocellular carcinoma: subanalyses of a phase III trial. J Hepatol. 2012;57:821–829 22727733 10.1016/j.jhep.2012.06.014 PMC12261288

[24] Vogel A, Qin S, Kudo M, Su Y, Hudgens S, Yamashita T, et al. Lenvatinib versus sorafenib for first-line treatment of unresectable hepatocellular carcinoma: patient-reported outcomes from a randomised, open-label, non-inferiority, phase 3 trial. Lancet Gastroenterol Hepatol. 2021;6:649–658 34087115 10.1016/S2468-1253(21)00110-2

[25] Galle PR, Finn RS, Qin S, Ikeda M, Zhu AX, Kim TY, et al. Patient-reported outcomes with atezolizumab plus bevacizumab versus sorafenib in patients with unresectable hepatocellular carcinoma (IMbrave150): an open-label, randomised, phase 3 trial. Lancet Oncol. 2021;22:991–1001 34051880 10.1016/S1470-2045(21)00151-0

[26] Qin S, Chan SL, Gu S, Bai Y, Ren Z, Lin X, et al. Camrelizumab plus rivoceranib versus sorafenib as first-line therapy for unresectable hepatocellular carcinoma (CARES-310): a randomised, open-label, international phase 3 study. Lancet. 2023;402:1133–1146 37499670 10.1016/S0140-6736(23)00961-3

[27] Doycheva I, Thuluvath PJ. Systemic therapy for advanced hepatocellular carcinoma: an update of a rapidly evolving field. J Clin Exp Hepatol. 2019;9:588–596 31695249 10.1016/j.jceh.2019.07.012 PMC6823698

[28] Kudo M, Finn RS, Qin S, Han KH, Ikeda K, Piscaglia F, et al. Lenvatinib versus sorafenib in first-line treatment of patients with unresectable hepatocellular carcinoma: a randomised phase 3 non-inferiority trial. Lancet. 2018;391:1163–1173 29433850 10.1016/S0140-6736(18)30207-1

[29] Saitoh M. Transcriptional regulation of EMT transcription factors in cancer. Semin Cancer Biol. 2023;97:21–29 37802266 10.1016/j.semcancer.2023.10.001

[30] Mir N, Jayachandran A, Dhungel B, Shrestha R, Steel JC. Epithelial-to-mesenchymal transition: a mediator of sorafenib resistance in advanced hepatocellular carcinoma. Curr Cancer Drug Targets. 2017;17:698–706 28460616 10.2174/1568009617666170427104356

[31] Faivre S, Rimassa L, Finn RS. Molecular therapies for HCC: looking outside the box. J Hepatol. 2020;72:342–352 31954496 10.1016/j.jhep.2019.09.010

[32] Eun JW, Yoon JH, Ahn HR, Kim S, Kim YB, Lim SB, et al. Cancer-associated fibroblast-derived secreted phosphoprotein 1 contributes to resistance of hepatocellular carcinoma to sorafenib and lenvatinib. Cancer Commun (Lond). 2023;43:455–479 36919193 10.1002/cac2.12414 PMC10091107

[33] Tan G, Xie B, Yu N, Huang J, Zhang B, Lin F, et al. TRIM37 overexpression is associated with chemoresistance in hepatocellular carcinoma via activating the AKT signaling pathway. Int J Clin Oncol. 2021;26:532–542 33387087 10.1007/s10147-020-01832-5

[34] Cai W, Ma Y, Song L, Cao N, Gao J, Zhou S, et al. IGF-1R down regulates the sensitivity of hepatocellular carcinoma to sorafenib through the PI3K / akt and RAS / raf / ERK signaling pathways. BMC Cancer. 2023;23:87 36698167 10.1186/s12885-023-10561-7 PMC9875405

