# Supplementary material for: CBX1 is involved in hepatocellular carcinoma progression and resistance to sorafenib and lenvatinib via IGF-1R/AKT/SNAIL signaling pathway
Source: Hepatol Int. 2024 May 20;18(5):1499–515. doi: 10.1007/s12072-024-10696-0 (PMC11461582; doi:10.1007/s12072-024-10696-0)
Supplement: Supplementary file 1 — Supplementary file1 (DOCX 5283 KB) [file 12072_2024_10696_MOESM1_ESM.docx]

Supplementary figure caption

Figure S1. Single-cell sequencing indicated CBX1 gene expression in HCC tissue. Cancer-associated fibroblasts (CAF).

Figure S2. CBX1 expression in liver tissue from the HPA dataset.

Figure S3. Gene Set Enrichment Analysis (GSEA) Revealing Activation of PI3K/AKT Pathway

Figure S4. Validation of Gene Knockout or Overexpression in HCC Cell Lines. (A) Snail gene knockout in HCCLM3 cell line. (B) Snail gene overexpression in MHCC97L cell line. (C) IGF-1R gene knockout in MHCC97L cell line. (D) IGF-1R gene overexpression in HCCLM3 cell line.

Figure S5. IGF1 expression in CBX1 knockout or overexpression HCC cells. (A/B) RT-PCR method for detecting the expression of IGF1 in HCC cells. (C/D) ELISA method for detecting the expression of IGF1 in the supernatant of cultured HCC cells.

Figure S6. Simplified diagram of the present study.

Figure S7: In Vitro and In Vivo Impact of CBX1 on HCC Progression. (A) CBX1 knockout validation in MHCC97H cells. (B) Confirmation of CBX1 overexpression in HepG2 cells. (C) Cell proliferation assay after CBX1 gene knockout in MHCC97H cells. (D) Cell proliferation assay after CBX1 gene overexpression in HepG2 cells. (E) Colony-Forming Assay after CBX1 gene knockout in MHCC97H cells. (F) Colony-Forming Assay after CBX1 gene overexpression in HepG2 cells. (G) Transwell invasion assay after CBX1 gene knockout in MHCC97H cells. (H) Transwell invasion assay after CBX1 gene overexpression in HepG2 cells. (I) CCK8 assays show TKI sensitivity in CBX1 knockout or overexpression cells. (J) Expression of EMT-related markers in MHCC97H cells after CBX1 gene knockout. (K) Expression of EMT-related markers in HepG2 cells after CBX1 gene overexpression. (L/M) Growth of subcutaneous HCC xenografts with altered CBX1 expression after 5 weeks. EV , empty vector.

Figure S8: CBX1, EMT, and TKI Resistance via AKT/SNAIL Signaling. (A) Western blot revealed the expression of phosphorylated AKT protein in HCC cells with different levels of CBX1. (B) EMT marker and AKT protein expression in HCC cells with different CBX1 levels. (C/D) Cell proliferation assay evaluated the proliferation in indicated HCC cells. (E/F) Transwell invasion assay evaluated the invasion ability in indicated HCC cells. (G/H) CCK8 assays showed TKI sensitivity in indicated HCC cells.

Figure S1 Single-cell sequencing indicated CBX1 gene expression in HCC tissue.


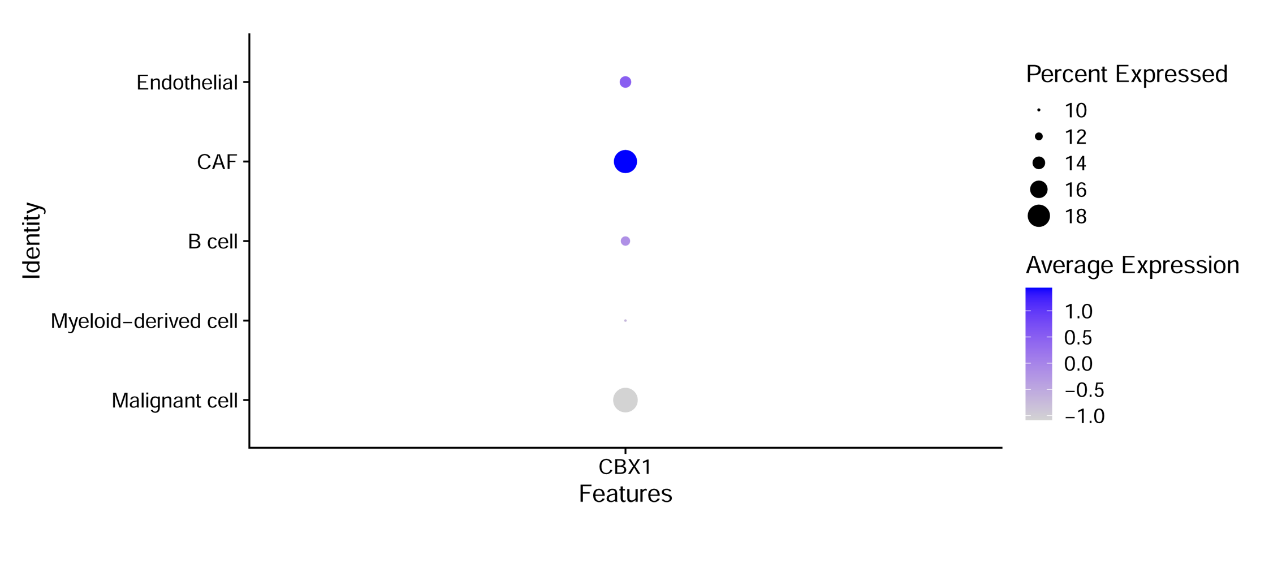


Figure S2 CBX1 expression in liver tissue from the HPA dataset.


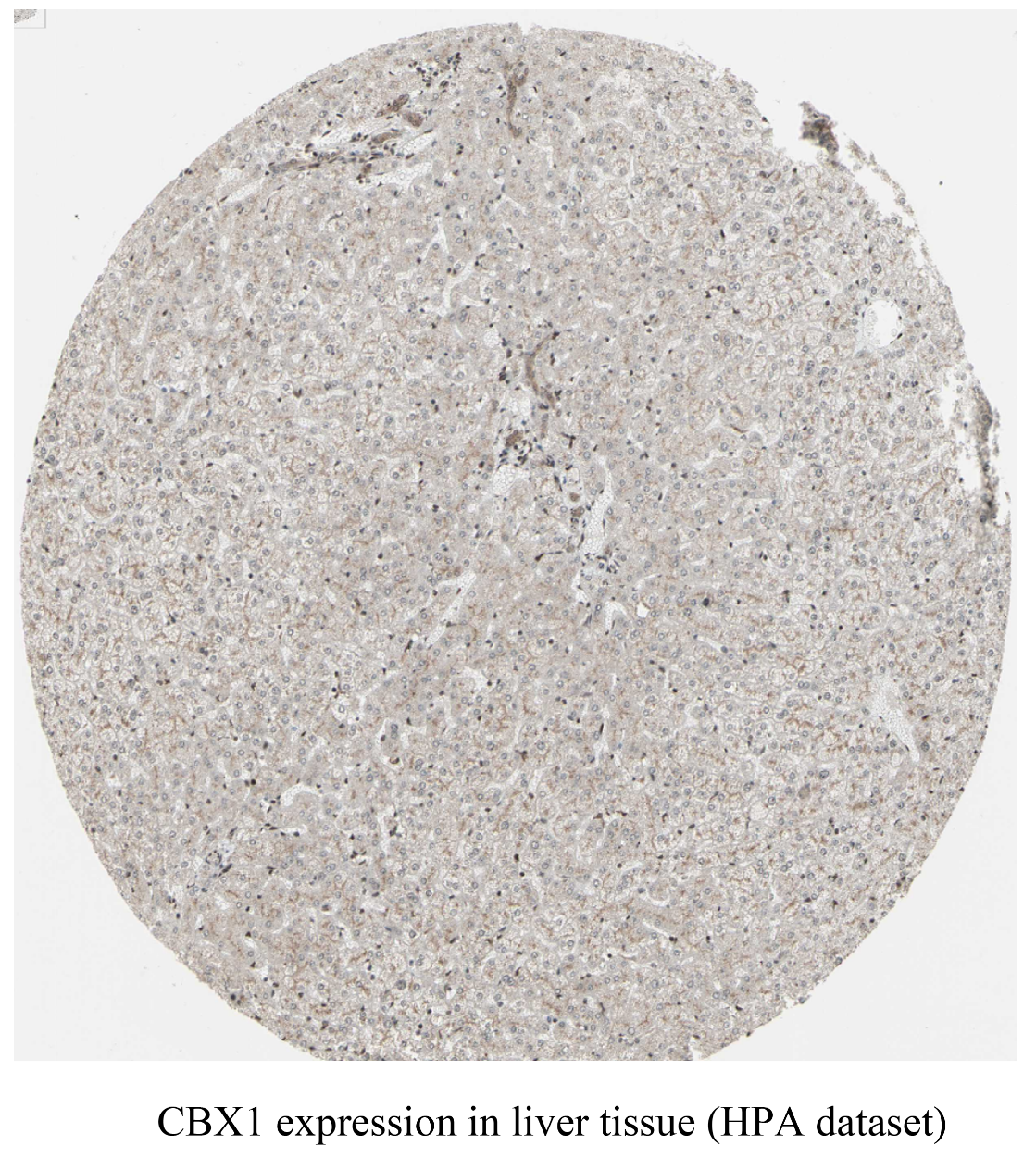


Figure S3 GSEA Revealing Activation of PI3K/AKT Pathway


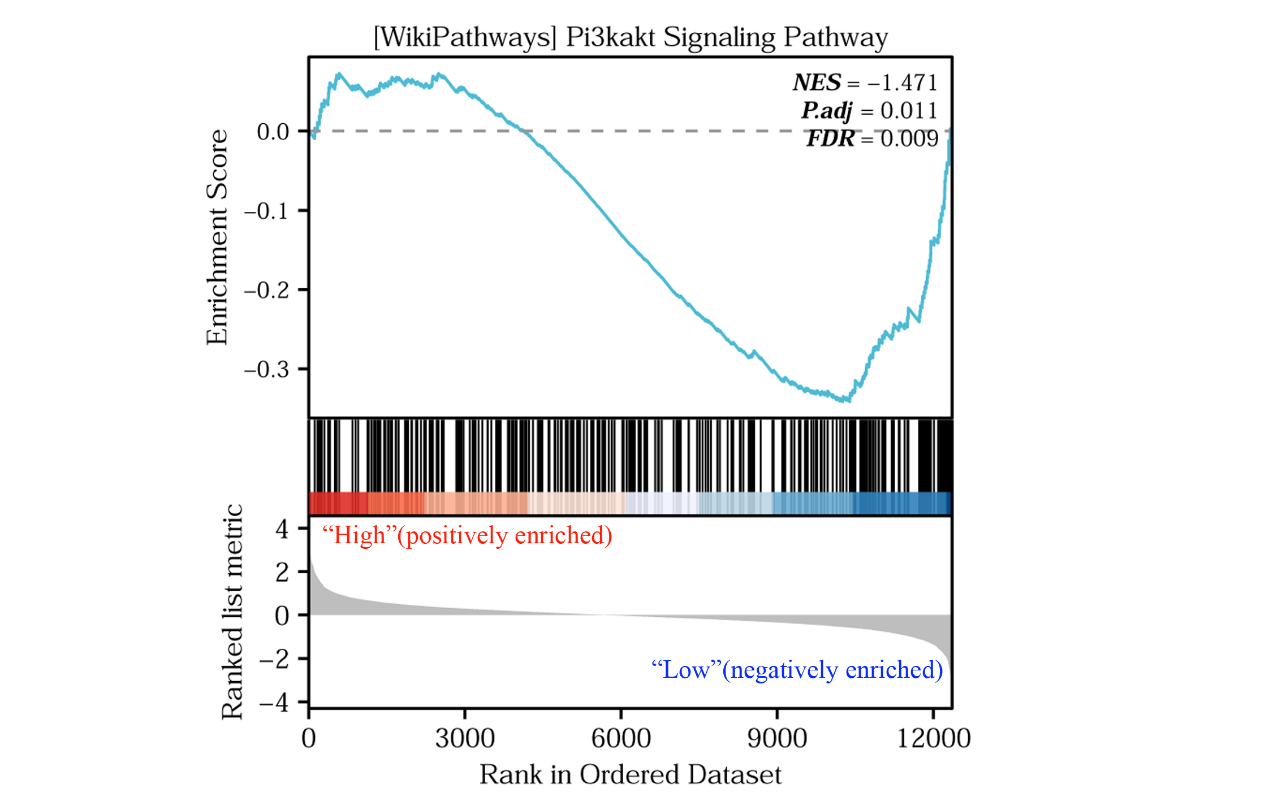


Figure S4 Validation of Gene Knockout or Overexpression in HCC Cell Lines.


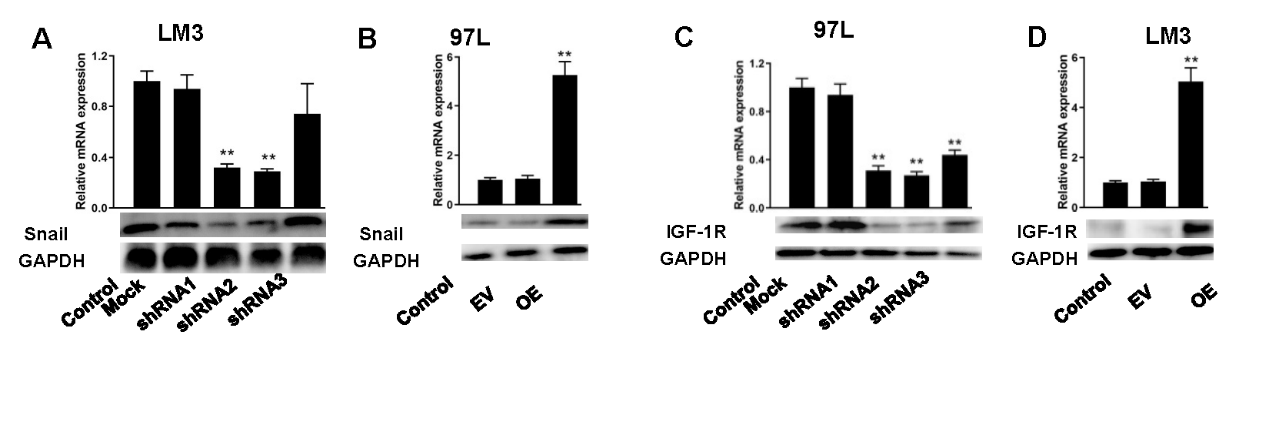


Figure S5 IGF1 expression in CBX1 knockout or overexpression HCC cells.


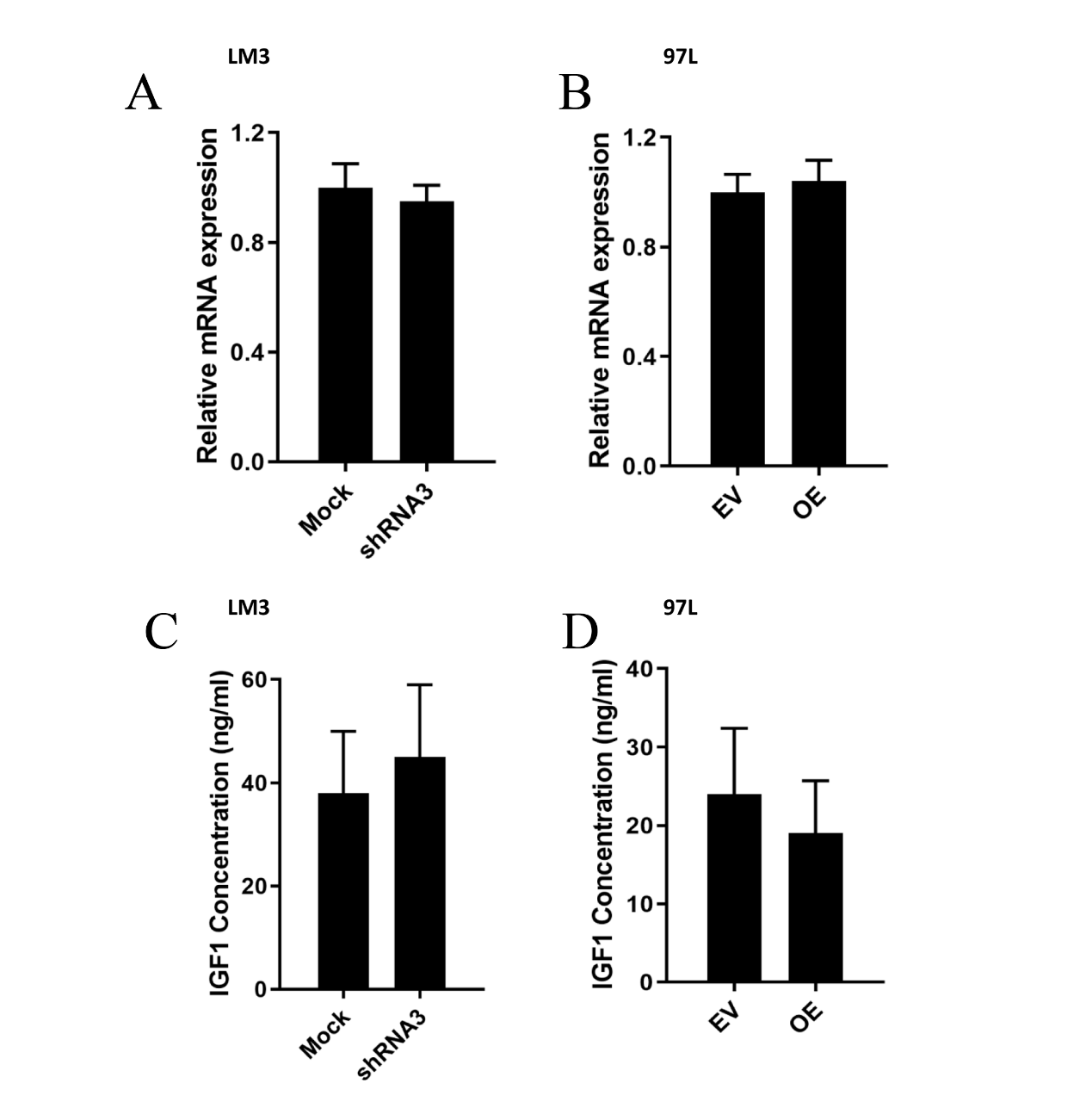


Figure S6 Simplified diagram of the present study.


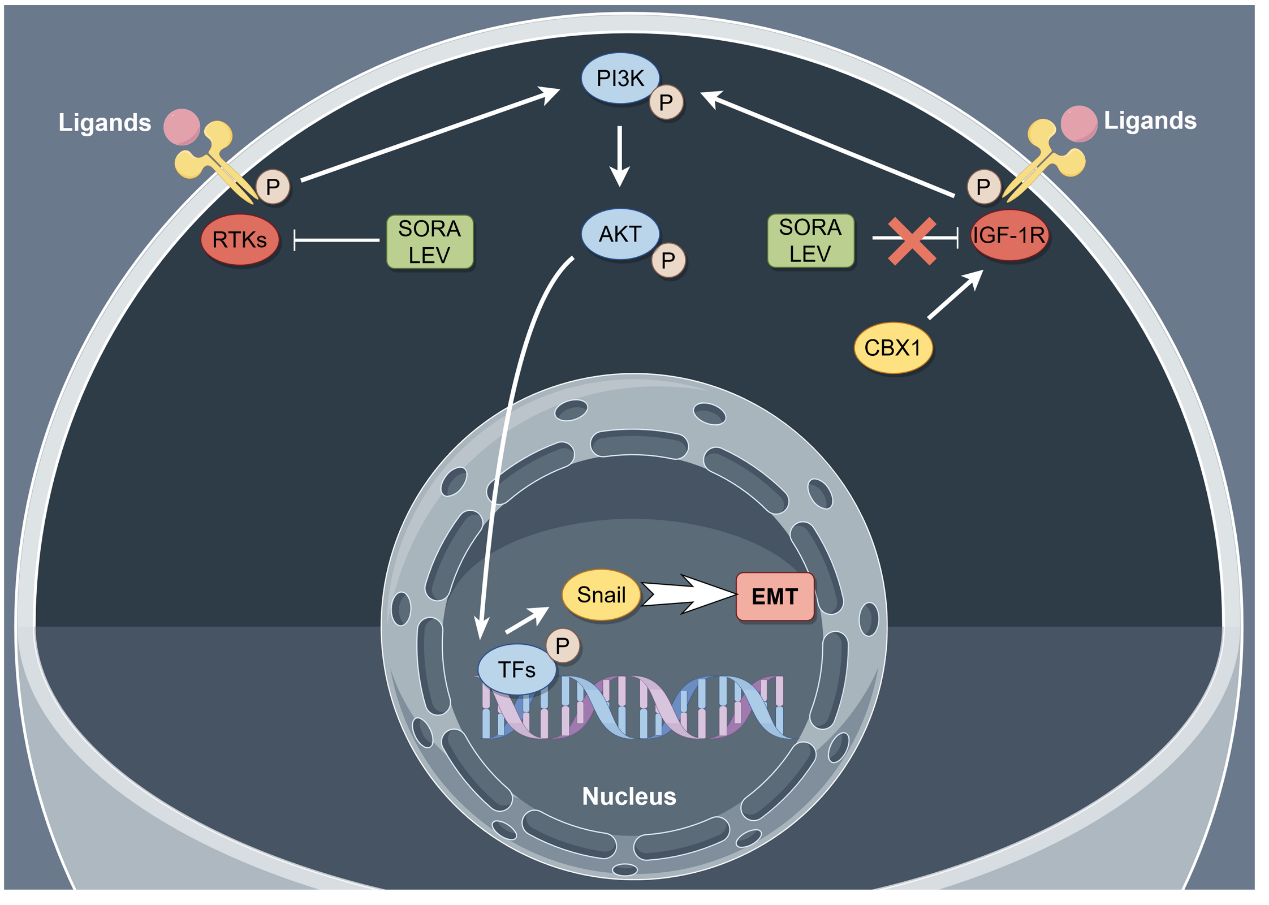


Figure S7: In Vitro and In Vivo Impact of CBX1 on HCC Progression.


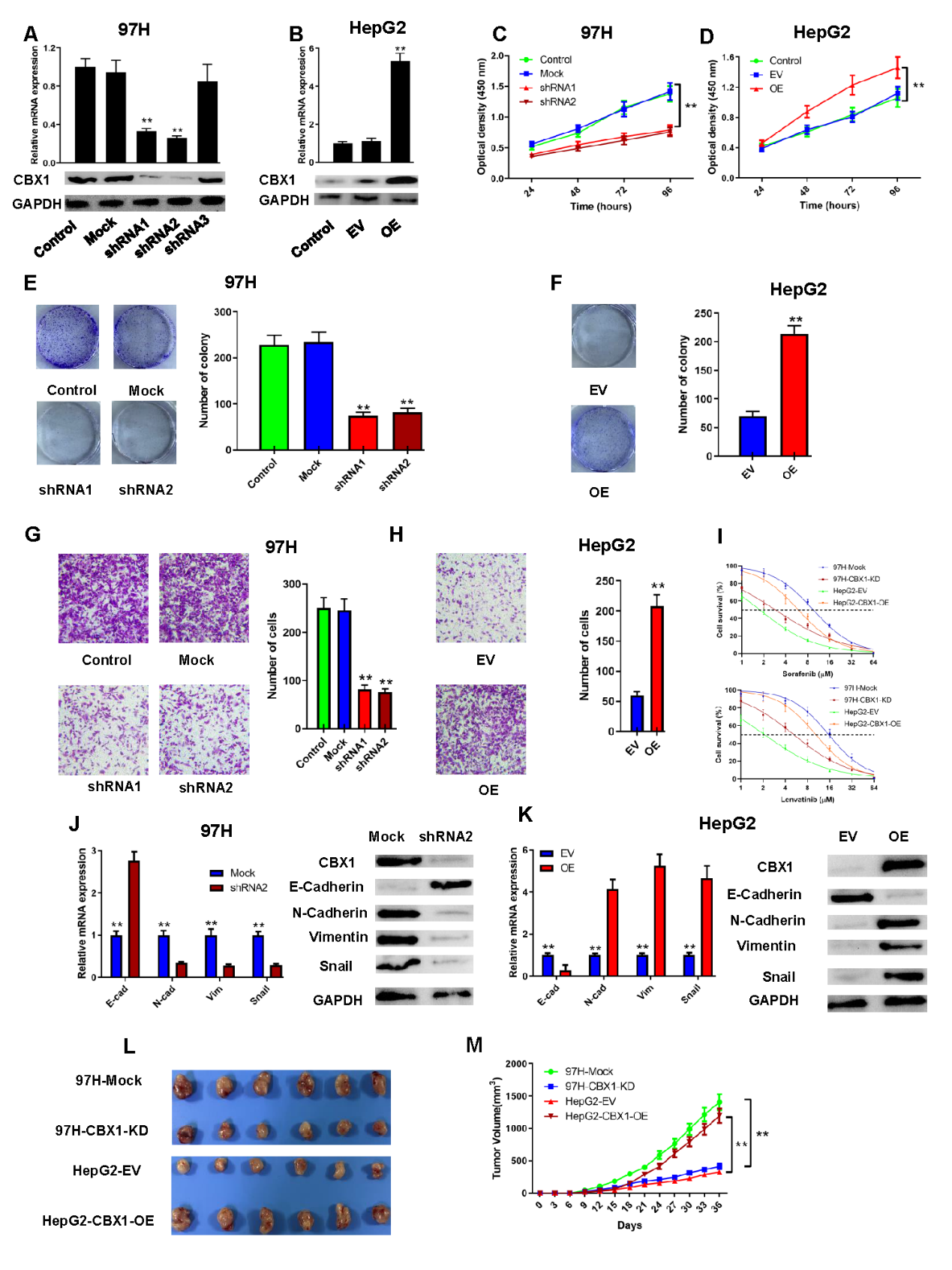


Figure S8: CBX1, EMT, and TKI Resistance via AKT/SNAIL Signaling.


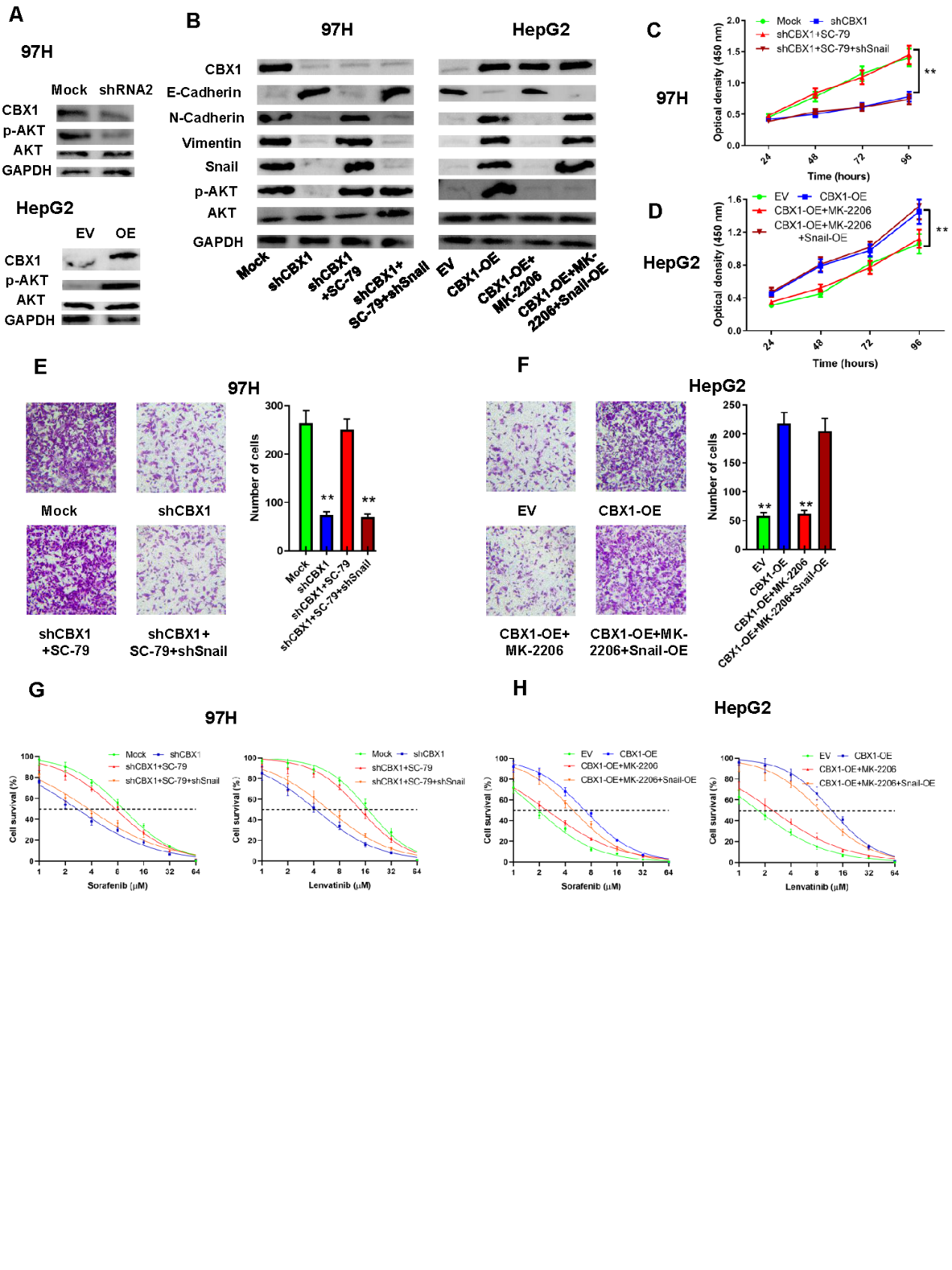


Supplementary Table 1. Primers used in this study for RT-PCR assays

| Gene | Forward primer (5'-3') | Reverse primer 5'-3' |
| --- | --- | --- |
| CBX1 | GGTGGAAAAAGTTCTCGACCG | CCCATGTGTTGTCCTCATCTG |
| IGF-1R | TCGACATCCGCAACGACTATC | CCAGGGCGTAGTTGTAGAAGAG |
| IGF-1 | TGTCCTCCTCGCATCTCTTCTACC | CCTGTCTCCACACACGAACTGAAG |
| Twist | GCCGACGACAGCCTGAGCAA | CGCCACAGCCCGCAGACTTC |
| Slug | CGAACTGGACACACATACAGTG | CTGAGGATCTCTGGTTGTGGT |
| Zeb1 | GATGATGAATGCGAGTCAGATGC | GAAGCGGTCATTCAGCTCCT |
| SNAIL | TCGGAAGCCTAACTACAGCGA | AGATGAGCATTGGCAGCGAG |
| vimentin | GACGCCATCAACACCGAGTT | CTTTGTCGTTGGTTAGCTGGT |
| E-Cadherin | CGAGAGCTACACGTTCACGG | GGGTGTCGAGGGAAAAATAGG |
| N-Cadherin | TCAGGCGTCTGTAGAGGCTT | ATGCACATCCTTCGATAAGACTG |
| Twist | GCCGACGACAGCCTGAGCAA | CGCCACAGCCCGCAGACTTC |
| EGFR | AGGCACGAGTAACAAGCTCAC | ATGAGGACATAACCAGCCACC |
| MET | AGCAATGGGGAGTGTAAAGAGG | CCCAGTCTTGTACTCAGCAAC |
| VEGFR1 | TTTGCCTGAAATGGTGAGTAAGG | TGGTTTGCTTGAGCTGTGTTC |
| FGFR1 | CCCGTAGCTCCATATTGGACA | TTTGCCATTTTTCAACCAGCG |
| KIT | CGTTCTGCTCCTACTGCTTCG | CCCACGCGGACTATTAAGTCT |
| PDGFR-α | TGGCAGTACCCCATGTCTGAA | CCAAGACCGTCACAAAAAGGC |
| TGFβ-R1 | ACGGCGTTACAGTGTTTCTG | GCACATACAAACGGCCTATCTC |
| GAPDH | GGAGCGAGATCCCTCCAAAAT | GGCTGTTGTCATACTTCTCATGG |
|  | | |
